# Insight into the Isoreticularity of Li-MOFs for the Design of Low-Density Solid and Quasi-Solid Electrolytes

**DOI:** 10.1021/acs.chemmater.3c01021

**Published:** 2023-11-28

**Authors:** Pravalika Butreddy, Manoj Wijesingha, Selina Laws, Gayani Pathiraja, Yirong Mo, Hemali Rathnayake

**Affiliations:** Department of Nanoscience, Joint School of Nanoscience & Nanoengineering, University of North Carolina at Greensboro, 1907 East Gate City Blvd, Greensboro, North Carolina 27401, United States

## Abstract

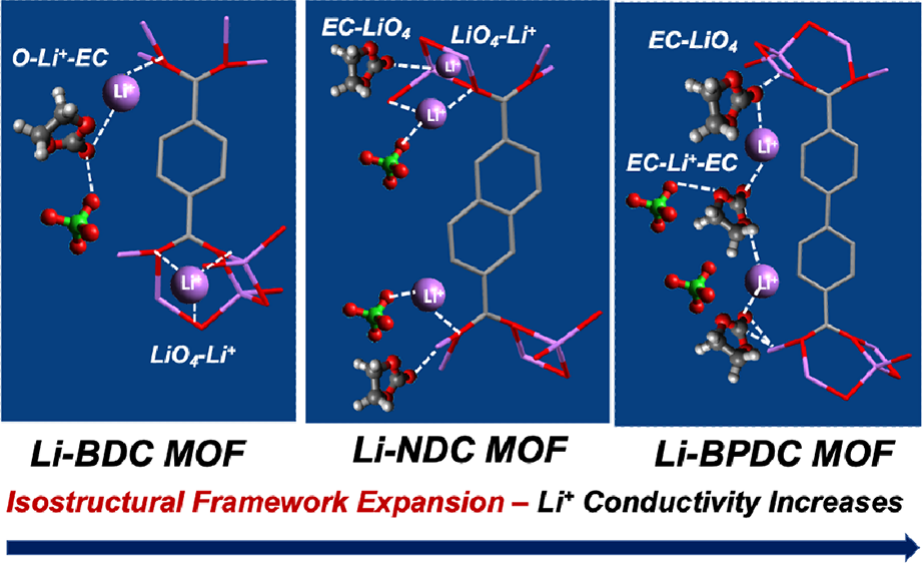

Isoreticularity in metal organic frameworks (MOFs) allows the design of the framework structure and tailoring the pore aperture at the molecular level. The optimal pore volume, long-range order of framework expansion, and crystallite size (grain size) could enable improving Li-ion conduction, thereby providing a unique opportunity to design high-performance solid and quasi-solid electrolytes. However, definitive understanding of the pore aperture, framework expansion, and crystallite size on the Li-ion conduction and its mechanism in MOFs remains at the exploratory stage. Among the different MOF subfamilies, Li-MOFs created by the isoreticular framework expansion using dicarboxylates of benzene, naphthalene, and biphenyl building blocks emerge as low-density porous solids with exceptional thermal stability to study the solid-state Li^+^ transport mechanisms. Herein, we report the subtle effect of the isoreticularity in Li-MOFs on the performance of solid and quasi-solid-state Li^+^ conduction, providing new insight into Li^+^ transport mechanisms in MOFs for the first time. Our experimental and computational results show that the reticular design on an isostructural extended framework structure with the optimal pore aperture and crystallite size can influence the Li^+^ conductivity, exhibiting comparable ionic conductivities to solid polymer electrolytes at room temperature. Aligning with the computational studies, our experimental absorption spectral traces of solid electrolytes prepared by encapsulating lithium salt (LiClO_4_) and the plasticizer (ethylene carbonate) with Li-MOFs confirm the participation of the free and bound states of Li^+^ in a pore filling-driven ion conduction mechanism. We postulate that porous channels of Li-MOFs aid free Li^+^ to move through the pores via a vehicle-type mechanism, in which the pore-filled plasticizer acts as a carrier for mobile Li^+^ while the framework’s functional sites transport the bound state of Li^+^ via an ion hopping mechanism from one crystallite site to another. Our computational studies performed on the Li^+^ conduction pathway validated the postulated pore filling mechanism and confirmed the involvement of bridging complexes, formed by binding Li^+^ onto the framework’s functional sites as well as to the pore-filled ethylene carbonates. The Li^+^ diffusion energy barrier profiles along with the respective conformational changes during the diffusion of Li^+^ in solid electrolytes prepared from Li-BDC MOF and Li-NDC MOF strongly support the cooperative movement of Li^+^ ions via ion hopping along the framework’s edges and vehicle-type transfer, involving the pore-filled plasticizer. Our findings suggest that cooperative function of the optimal pore volume, framework expansion, and crystallite size play a unique role in Li-ion conduction, thereby providing design guidelines for the low-density solid and quasi-solid electrolytes.

## 1 Introduction

A quest for next generation all solid-state lithium-ion batteries (SSLBs) with a higher energy density (>300 kWh/kg), longer life cycle (10–15 years), and acceptable thermal and mechanical stability has been accelerated over the past few decades due to the increasing energy demands from portable electronics to grid energy storage systems.^1,2^ A high-performance, lightweight solid-state electrolyte (SSE) is one of the most crucial components that holds the key to propel SSLBs into next generation energy storage technologies. Nonetheless, it is becoming increasingly urgent to study solid-state lithium-ion conduction by screening the electrochemical performance of “materials-in-demand” (i.e., having unprecedented chemical and structural tunability). This rapid analytical screening approach enables the discovery of the best potential design approach to overcome the poor performance of the current SSEs. Toward this goal, efforts should be devoted to understanding the structure–function dynamics of materials at the molecular level and tuning their function to be highly specific and cooperative for effective and efficient ion conduction.

Reticular chemistry provides ability to control the material’s properties at the molecular level by linking discrete building units (also called secondary building units, SBUs), via strong coordination bonds, yielding large and extended crystalline porous structures, called metal–organic frameworks (MOFs).^3−6^ Their synthetic versatility, long-range order, and rich host–guest chemistry make MOFs one of the most demanding materials for the design of advanced functional materials with optimized properties to combat inherent electrochemical limitations in SSEs. Owing to MOFs precisely positioned metal nodes by connecting with functional metal-carboxylate SBUs via coordination bonds, they allow for designing the framework structure and tailoring the pore environment at the molecular level.^7^ With creative reticular synthetic design approaches, MOFs’ properties such as porosity, stability, structural morphology, and conductivity can be tailored for a specific application. For instance, the pore aperture itself plays a crucial role in host–guest chemistry of MOFs by minimizing the diffusion kinetics of guest molecules and thereby facilitating selective ion transport through the porous network.^8,9^ The structural diversity encountered in the chemistry of the MOF originates largely from a wide variety of accessible metal-carboxylate SBU geometries. Thus, it is possible to target functionality and pore aperture-tuned porous structures by choosing appropriately shaped and sized building units.^10^ The design of extended framework of MOFs with different topological structures from a variety of molecular building units has been revealed by establishing the reticular chemistry toolbox.^4−6^ The reticular design principles of the SBU approach offer a pathway to making isoreticular MOFs (IRMOFs). They are isostructural extended frameworks constructed by the expansion of the spacing between vertices in a net with the replacement of different lengths of organic linkers without changing the topological structure and geometry of the SBU. In principle, the reticular expansion should yield the frameworks with wider pore aperture dimensions, but in practice, they often result in a highly interpenetrated structure with either low porosity or a narrow pore aperture.^7,10^ The IRMOF series with the formula of Zn_4_O(L)_3_ (where L is a rigid linear dicarboxylates) based on the topological structure of MOF-5 (IRMOF-1) is one of the outstanding examples for the pore aperture-tuned MOFs reported by Yaghi et al.^11^ It has demonstrated that, when such networks are interpenetrating, optimal pore aperture can be achieved to uptake charged ionic species, minimizing the activation energy and increasing the ionic conductivity.^3,7,10,12−21^

An increasing number of MOFs has been extensively tested as a solid-state and quasi-solid-state for proton conductance with conductivity values varying in a wide range of 10^–5^ to 10^–2^ S cm^–1^.^15−17,22−26^ A few MOFs have studied for the conductance of small alkali cations, like Li^+^, Na^+^, and K^+^, by introducing these ions into the framework, following common strategies of: (1) post synthetic modification of the SBUs, augmenting open metalation concept (OMC)^27,28^ to obtain anionic framework,^12,13,18−20^ and (2) host–guest encapsulation, which mainly relies on the loading capacity of the pore structure (aperture size and the functionality) of MOFs.^12−14,18−21^ The postmodified anionic framework balances the charge with freely mobile Li^+^ ions, allowing to move freely in the one-dimensional channels.^13,20^ For example, Wiers et al. reported post synthetic grafting of open metalation sites (OMSs) in Mg-MOF (Mg_2_(dobdc), dobdc = 2,5-dioxido-1,4-benzenedicarboxylate), with LiO^*i*^Pr and demonstrated that it could serve as an ideal Li-ion conductor at room temperature, yielding an ionic conductivity of 3.1 × 10^–4^ S cm^–1^ with an activation energy (*E*_a_) of only 0.15 eV.^12^ Subsequently, the effect of OMSs and pore sizes of MOFs on the Li^+^ ion conductance was revealed using different MOFs, such as MOF-5, HKUST-1, MIL-100(Al, Cr, or Fe), UIO-66, and UIO-67.^13^ It was demonstrated that the ionic conductivity varies depending on the metal type of OMS and that there is a clear effect of the pore size on the ionic conductivity. The results indicate that larger pore size could make Li^+^ solvation more effectively and reduce the confining effect, yielding high ionic conductivity.^13^ Besides, two neutral isostructural Li-based MOFs (Li-AOIA and Li-TMCA), which belong to the *C*2/*c* space group, were explored for their potential use as SSEs, by successive doping of LiBF_4_ into the activated Li-MOF (Li-AOIA@BF_4_ and Li-TMCA@BF_4_) through modification of its open metal sites. Li-AOIA@BF_4_ showed the highest ionic conductivity of 1.09 × 10^–5^ S cm^–1^ with an activation energy of 0.18 eV at room temperature.^14^ Although these low-density Li-MOFs exhibit rather low ionic conductivity, compared to previously described MOFs,^13,29^ this work sheds light on developing low-density solid-state and quasi-solid-state electrolytes from Li-based MOFs.

For the first time, herein, we reveal the effect of isoreticularity on the fundamental understanding of lithium-ion conduction in three isoreticular Li-MOFs, providing an effective analytical approach for the design of high-performance solid and quasi-solid electrolytes. These synthetically known three isoreticular Li-MOFs, Li-BDC MOF, Li-NDC MOF (i.e., originally known as ULMOF-1), and Li-BPDC MOF (i.e., originally known as ULMOF-2), are constructed by linking LiO_4_ metal oxide nodes (SBUs) with organic subunits of benzene-1,4-dicarboxylic acid (BDC), 2,6-naphthalene dicarboxylic acid (NDC), and 4,4′-biphenyl dicarboxylic acid (BPDC), respectively. Even though enormous scientific significance of the MOF-based electrolytes has been established, insight into reticular design on the isostructural extended framework structure with the optimal pore aperture and crystallite size (grain size) in Li-MOFs has not been investigated to recognize their cooperative function on lithium-ion conductivity and especially its mechanism in the solid state. In this context, we have investigated the lithium-ion conduction in isoreticular Li-MOFs and revealed their Li^+^ ion conduction mechanisms with the aid of computational methods. We were able to deduce the profound effect of the isoreticularity on the Li-ion conduction by understanding the function of their extended framework, crystalline size, porosity distribution, and the associated host–guest chemistry. Our study established that isoreticular Li-MOFs could be a potential candidate for designing low-density, high-performance solid and quasi-solid electrolytes.

## 2 Materials and Methods

### Materials

Lithium nitrate (LiNO_3_) was purchased from Honeywell. Benzene-1,4-dicarboxylic acid (1,4-BDC, 98% purity), 2,6-naphthalenedicarboxylic acid (2,6-NDC, 95% purity), biphenyl-4,4′-dicarboxylic acid (4,4′-BPDC, 97% purity), lithium perchlorate (LiClO_4_; molar mass = 106.39 g/mol), ethylene carbonate (EC: C_3_H_4_O_3_; molar mass = 888.06 g/mol), anhydrous ethanol (200 proof), and *N*,*N*-dimethylformamide (DMF: anhydrous, 99.8%) were obtained from Sigma-Aldrich. All chemicals were used as received, unless otherwise specified.

### Characterization

The chemical composition, functional groups, and their binding interactions were analyzed using Fourier transform infrared spectroscopy (FTIR-Varian 670-IR spectrometer). The elemental composition and chemical oxidation states of the elements were obtained from X-ray photon spectroscopy (XPS-Escalab Xi+-Thermo Scientific). The microelemental composition was matched with the XPS elemental survey analysis. The powder XRD analysis was conducted using Cu Kα radiation (40 kV, 40 mA, *k* = 1.540 Å) with a speed of 90 s on the X-ray diffractometer (XRD, Agilent technologies Gemini). The HR-TEM (JEOL 2100PLUS) with a STEM/EDS capability was used to analyze Li-MOF microstructures’ morphologies and crystallinity, including lattice parameters, local framework structure, and selective area diffraction patterns. The thermal stabilities of microstructures were analyzed by using a thermogravimetric analyzer (Q500). Samples were heated up to 1000 °C from an initial temperature of 22 °C at the increment of 5 °C/min under the nitrogen flow. Full N_2_-isotherm with porosity analysis for the Brunauer–Emmett–Teller (BET) surface area, Barrett–Joyner–Halenda (BJH) adsorption/desorption isotherms, BJH adsorption and desorption cumulative pore volumes and cumulative pore area distributions using t-plots, and pore width and pore volume distributions using nonlocal density functional theory (NLDFT) and Horvath–Kawazoe Cumulative Pore Volume Plots were performed using the Micrometrics analyzer accelerated surface area and porosimetry (ASAP #2060) system by Micromeritics Instrument Corp. BJH adsorption–desorption data were subjected to the Chi-square goodness of fit test to determine the adsorption and desorption isotherms in the micropore region, representing the full distribution of data collected for BJH isotherms. In a typical analysis method, a powder sample of Li-MOF (200 mg) was packed in a BET sample tube. The sample was degassed at 90 °C for 1 h followed by additional 12 h at 250 °C to remove any moisture and atmospheric gases occupied in the accessible pores. After the postdegassing, the sample weight was recorded before analysis for accurate results. Full N_2_-adsorption/desorption isotherms were measured at 77 K. The low-pressure incremental dose amount was 3.0 cm^3^/g STP with equilibration intervals 40, 30, and 20 s.

### Synthesis of Isoreticular Li-MOFs

All three Li-MOFs were synthesized using a modified solvothermal method, adapted from the previously reported synthesis methods.^29−31^ Our modified solvothermal method used lithium nitrate as the metal precursor, and the solvothermal process was performed in dimethylformamide at 110 °C for 3 days in a capped vial using a sand bath to yield Li-BDC MOF as colorless needle-like crystals, Li-NDC MOF as a yellow powder, and Li-BPDC MOF as a white powder. In a typical procedure, three Li-MOFs were synthesized by maintaining the metal precursor (0.69 g, 0.010 mol) to the organic linker molar ratio at 2:1 and by simply changing the organic linker to benzene-1,4-dicarboxylic acid (BDC, 0.83 g, 0.005 mol), 2,6-naphthalene dicarboxylic acid (NDC, 1.08 g, 0.005), and biphenyl-4,4′-dicarboxylic acid (BPDC, 1.21 g, 0.005 mol) to yield Li-MOFs of Li-BDC, Li-NDC, and Li-BPDC, respectively. The product (yields: 28, 37, and 27%, respectively) was collected by washing with cold anhydrous DMF and vacuum drying at 250 °C overnight to remove any residual surface-adsorbed moisture and DMF. The structural and composition analyses were performed and confirmed their crystal structures from powder XRD analysis combined with simulated XRD spectra obtained for original crystal structures of Li-BDC MOF from the Cambridge Crystallographic Data Center (deposition #: CCDC 664607) and from cif files of ULMOF-1 and ULMOF-2.^30,31^ The crystal structures were visualized by using VESTA (version 3.5.8).

### FTIR Stretching (ν, cm^–1^)

Li-BDC MOF: 1570 (metal-ion-coordinated carbonyls), 1501 (aromatic C=C), 1390 (sharp peak, Li–O–C–O stretching), 1095 (C–O), 825 (Li–O stretching), 750 (aromatic C=C); elemental compositional analysis for the empirical formula of Li_2_C_8_H_4_O_4_: experimental—C (55.98), Li (7.87), and O (36.15); theoretical—C (53.98), Li (7.80), O (35.95), and H (2.27); Li-NDC MOF—1603-1570 (metal-ion-coordinated carbonyls), 1500 (aromatic C=C), 1392 (sharp peak, Li–O–C=O), 802 (Li–O), 778 (aromatic C=C); elemental compositional analysis for the empirical formula of Li_2_C_12_H_6_O_4_: experimental—C (64.03), Li (6.05), and O (29.92); theoretical, C (63.20), Li (6.09), O (28.06), and H (2.65); Li-BPDC MOF, 1591 (metal-ion-coordinated carbonyls), 1538 (aromatic C=C), 1397 (sharp peak, Li–O–C=O), 842 (Li–O), 772 (aromatic C=C); elemental compositional analysis for the empirical formula of Li_2_C_14_H_8_O_4_: experimental—C (64.03), Li (6.05), and O (29.92); theoretical—C (63.20), Li (6.09), O (28.06), and H (2.65).

### Preparation of LEC@Li-MOF Pellets

The power samples of Li-MOFs (300 mg, thermally treated at 250 °C) were soaked in the LEC solution (5 wt % LiClO_4_ in 2g EC, 2.1 g of solid) and heated at 80 °C. Then, the powder was collected by filtering the excess liquid using a Whatman filter at 80 °C and dried at room temperature in a vacuum oven over 72 h to yield yellow powder (∼600 mg). The dry LEC@Li-MOFs powder (∼600 mg) was pelletized at 44 MPa on a hydraulic press to yield solid pellets with a diameter of 1.5 cm and a thickness of 1.2–2.0 mm. The weight of LEC incorporated in the dry powder of LEC@Li-MOFs was determined to be ∼300 mg. From the XPS elemental survey analysis, the percent weight of excess lithium-ion retained was found to be ∼1.5% for all the samples. The percent weight of oxygen was increased by ∼10% for LEC@Li-BDC and LEC@Li-NDC samples and by ∼20% for LEC@Li-BPDC pellets, confirming the percent weight of EC retained in the pellets.

### Ionic Conductivity Measurements

Nyquist plots of pellets prepared from each LEC@Li-MOF were collected by using electrochemical impedance spectroscopy (EIS) with a VMP3 Bio-Logic multichannel potentiostat. In a typical measurement setup, the pellet was sandwiched between two gold-coated copper disk (25 mm diameter) electrodes linked to an alternating current at 10 mV amplitude with a frequency range from 10^6^ to 10 Hz. The ionic conductivity (σ, S cm^–1^) was determined based on eq 1.
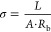
1where *R*_b_ is the bulk resistance obtained from the Nyquist plot, *L* is the thickness of the pellet, and *A* is the surface area of the pellet.

The temperature-dependent impedance spectra were recorded in the temperature range from 25 to 65 °C, repeated three times at each temperature in the air. Using the Arrhenius relation equation (eq 2), the activation energies were calculated from the slope of the graphs on the ln σ_(T)_ versus 1000/*T*.
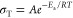
2where σ_T_ is ionic conductivity at a particular temperature (*T*), *A* is the pre-exponential factor, *E*_a_ is the activation energy, *R* is the universal gas constant, and *T* is the absolute temperature (in Kelvin).

### Computational Methodology

Density functional theory (DFT) methods were used to carry out the electronic structure calculations through the Perdew–Burke–Ernzerhof (PBE) exchange-correlation functional in the Vienna *Ab Initio* Simulation Package (VASP). The conjugate gradient (CG) algorithm was utilized for the structural relaxation and for the electron–ion interactions, and the projector augmented wave (PAW) method was applied. The kinetic energy cutoff was set to 500 eV for the plane-wave bases. The convergence criterion for the electronic structure iteration was 10^–5^ eV, and for geometry optimization, it was 0.01 eV/Å. A Monkhorst–Pack 3 × 5 × 3 *k*-point grids were adopted for the unit cells of three Li-MOFs and EC (ethylene carbonate). For the Li^+^-ion complexes of LEC@Li-BDC, LEC@Li-NDC, and LEC@ BPDC, the *k*-points were set to 1 × 2 × 1 since we employed the supercells for computational simulations. We identified Li^+^ ion diffusion pathways in the isoreticular Li-MOFs of Li-BDC and Li-NDC, but the pathway in Li-BPDC was unavailable due to the convergence issues in the structural energy minimizations. Density functional perturbation theory (DFPT) was employed for the phonon calculations with the matrix of Born effective charges (BEC),^32−38^ which was used to generate vibration intensities for the system. Li^+^ ion diffusion pathways in Li-MOFs were calculated with the climbing image nudged elastic band (CINEB) method.^39−42^ To obtain the energy profiles, the geometry of the initial and final systems were optimized first for the energy minimization, starting from the DFT calculations. By performing a linear interpolation between the initial and final states, we generated a set of images to obtain an approximation of the reaction path of the system. Finally, we performed a simultaneous optimization of all the images by the CINEB method to identify reaction coordinates in the reaction path of each respective system.

## 3 Results and Discussion

### Synthesis and Characterization of Isoreticular Li-MOFs

**Scheme 1:**
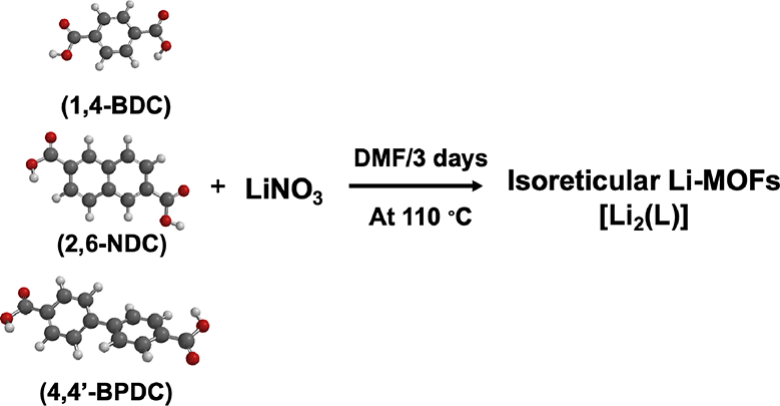
Synthetic Scheme for the Preparation of Isoreticular Li-MOFs

Adapting a previously reported solvothermal method,^29−31^ we have synthesized three isoreticular Li-MOFs, using LiNO_3_ as the metal precursor and anhydrous DMF as the solvent (Scheme 1). The prior reported solvothermal method used either LiNO_3_ or LiClO_4_ as the metal precursor in the presence of solvent mixtures of either DMF/ethylene glycol or DMF/NH_4_F.^29−31^ Isoreticular Li-MOFs prepared in this manner were confirmed by their elemental composition analysis using XPS and FTIR spectroscopy. Their chemical structures were confirmed by matching the experimental powder XRD traces with their respective simulated powder diffraction patterns acquired from their originally reported crystal structures. Their textural properties and porosity distribution were also elucidated from their N_2_ absorption–desorption analysis.

**Figure 1:**
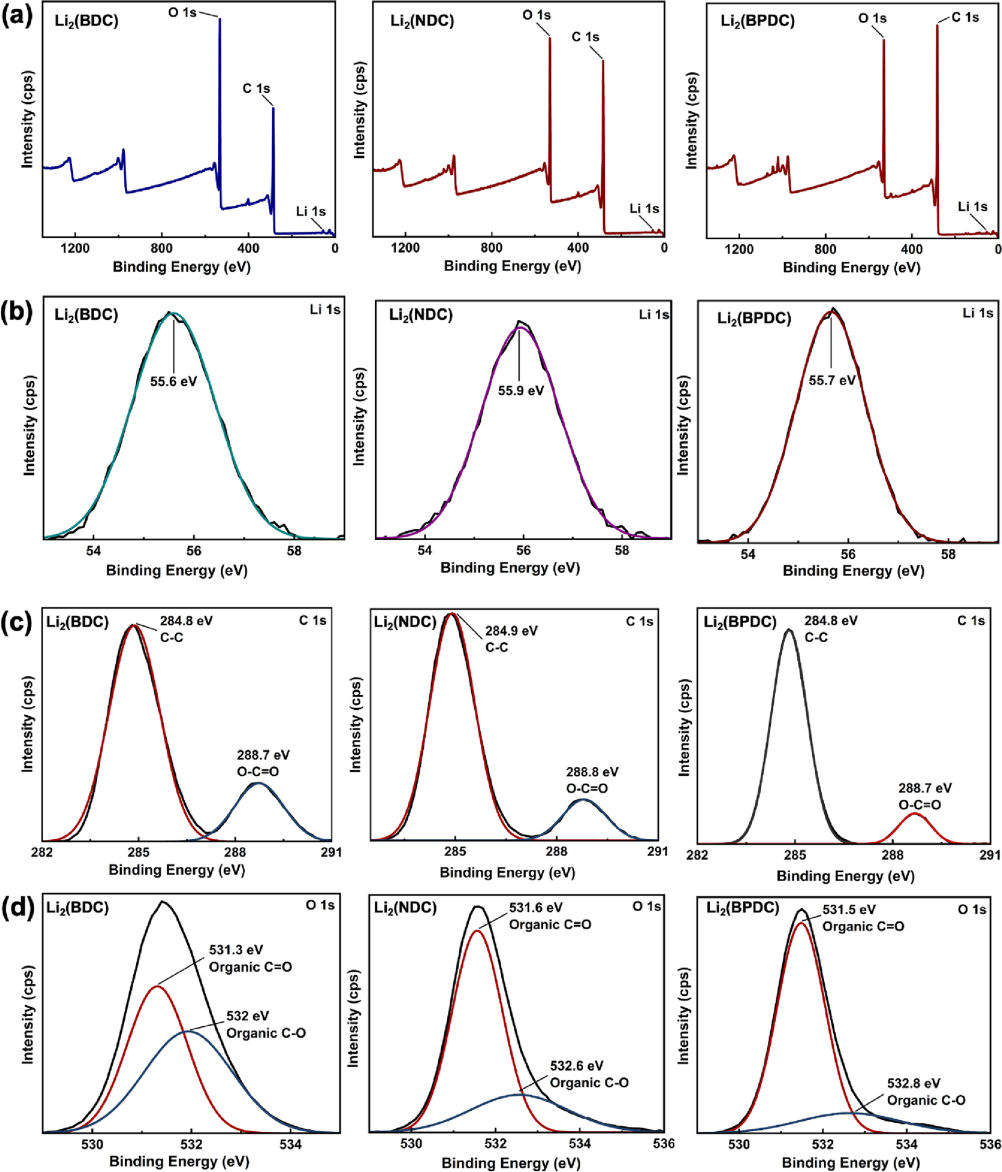
(a) XPS survey spectra and binding energy spectra of (b) Li 1s, (c) C 1s, and (d) O 1s for isoreticular Li-MOFs.

The XPS survey spectra along with the binding energy spectra corresponding to each element’s chemical bonding environments for three Li-MOF analogues, depicted in Figure 1, evidence the successful synthesis of three Li-MOF analogues with monovalent oxidation states of Li in LiO_4_ inorganic clusters. The XPS survey spectra of the three MOFs in Figure 1a confirm the presence of Li, C, and O with the absence of solvent impurities (DMF) in all three samples. The binding energies of Li 1s obtained for three MOFs confirm the lithium’s monovalent oxidation state with respect to their binding energy peaks at 55.6, 55.9, and 55.7 eV, respectively (Figure 1b.^43^ The deconvoluted C 1s spectra of Li-MOF analogues exhibit two binding energies at 284.8 and 288.8 eV for the chemical bonding states of sp^2^ C=C and O–C=O, respectively, (Figure 1c).^44^ The binding energies at 530.3 530.6, and 530.5 eV for the O 1s spectra for all three analogues, respectively, confirm the presence of carbonyl bonding. Additionally, the binding energies at 532.0, 532.6, and 532.8 eV confirm the formation of Li–O–C coordination bonds (Figure 1d).^43^Table S1 summarizes the elemental compositions obtained from the XPS elemental survey bulk analysis.

The experimental FTIR spectra of all three Li-MOFs (Figure S1) confirm the formation of the metal-carboxylate coordination framework from the stretching of carboxylate carbonyls at 1570, 1603–1570, and 1591 cm^–1^ for Li_2_(BDC), Li_2_(NDC), and Li_2_(BPDC), respectively.^44−47^ The carbonyl vibrations of organic linkers’ carboxylic acids are at 1670–1675 cm^–1^. Additionally, the successful coordination of Li-ions with carbonyl oxygen, forming LiO_4_ metal oxide nodes, was confirmed from the stretching at 1390–1397 and 802–847 cm^–1^, which correspond to Li–O–C–O and Li–O stretching, respectively. All three FTIR spectra lack a broad hydroxyl stretching around 3000–3300 cm^–1^, confirming the absence of surface-adsorbed water, agreeing with their elemental composition analysis, which confirms Li-MOF empirical formula to be Li_2_C_8_H_4_O_4_ (Li_2_(BDC)), Li_2_C_12_H_6_O_4_ (Li_2_(NDC)), and Li_2_C_14_H_8_O_4_ (Li_2_(BPDC)), respectively.

**Figure 2:**
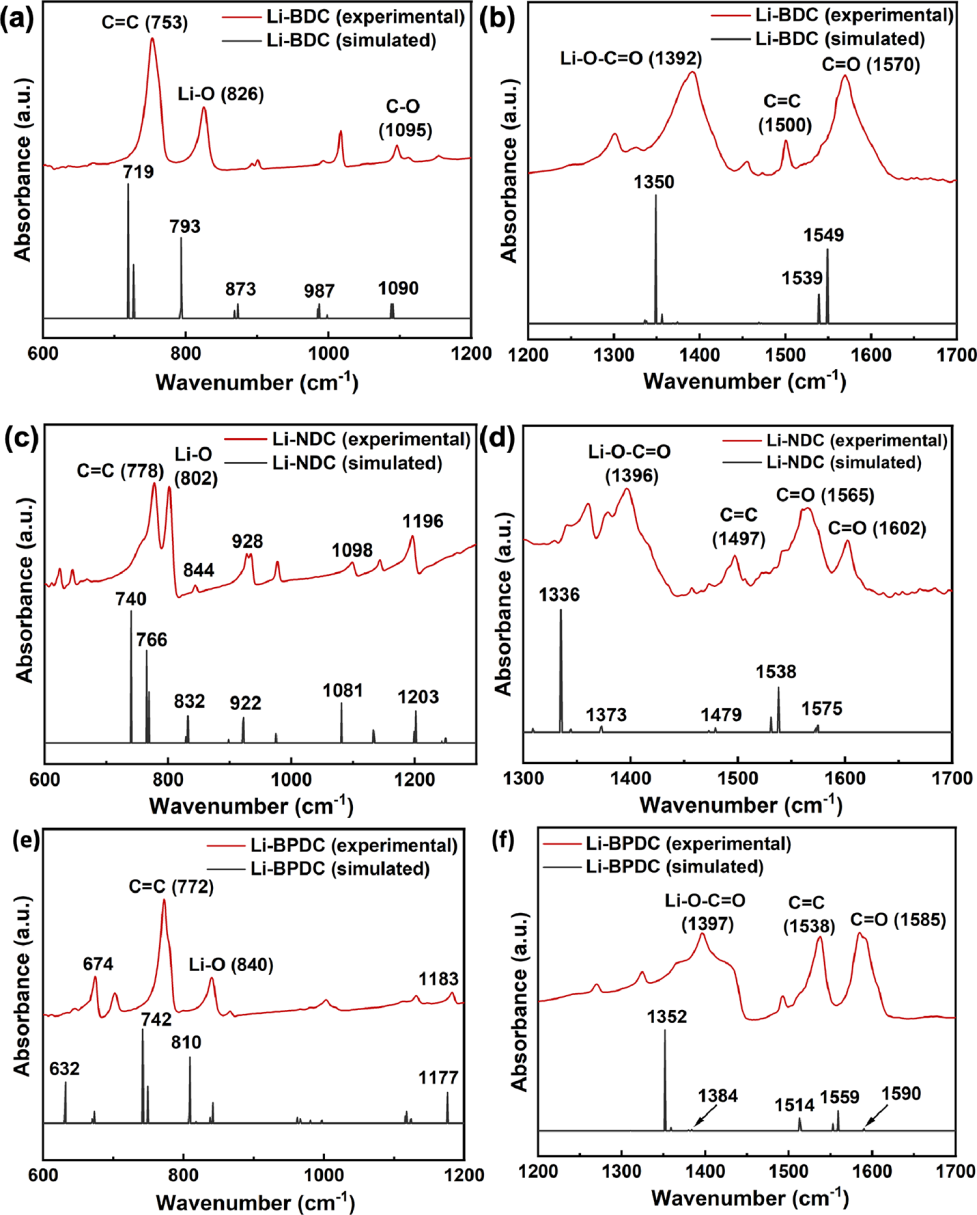
Experimental and simulated absorption spectra of Li-MOFs: (a, b) Li-BDC, (c, d) Li-NDC, and (e, f) Li-BPDC at wavenumber regions of 600–1200 and 1200–1700 cm^–1^.

The simulated FITR spectra obtained from the optimized crystal structures of Li-MOFs agree with the experimental spectral traces, yielding key vibronic stretching for the confirmation of the formation of three MOFs (Figure 2 and Figure S2). The key vibronic frequencies at ν = 719, 793, 1095, 1350, 1539, and 1549 cm^–1^ in the simulated absorption spectrum of the Li-BDC MOF confirm its framework (Figure 2a,b), agreeing with the respective experimental vibronic frequencies. Similarly, the main vibronic frequencies (ν = 740, 766, 1203, 1373, 1479, 1538, and 1575 cm^–1^) of the simulated absorption spectrum of the Li-NDC MOF (Figure 2c,d) align with the experimental absorption spectrum, validating the framework. The simulated absorption spectrum obtained for the optimized structure of Li-BPDC MOF reflects the framework functional groups, supporting the experimental FTIR spectrum’s vibronic frequency assignments. It is worth to mention that vibronic frequencies of the simulated FTIR spectral traces of all three Li-MOFs are somewhat shifted to lower frequencies compared to the experimental vibronic frequencies. The shift in simulated vibronic frequencies is typical as simulated FTIR spectra were computed from the respective crystal lattice vs the experimental spectral traces of bulk powder samples.

Thermal stabilities of Li-MOFs were analyzed by thermogravimetric analysis (Figure S3). The first degradation of the Li-BDC framework (Figure S3a) occurs at 551 °C with a weight loss of 46%, demonstrating that the framework is stable up to 551 °C. Thereafter, the framework starts to collapse and completely decomposes to char at 709 °C, yielding 26% as the final char yield, corresponding to the inorganic content, Li_2_O. The total weight loss of 74% occurs in the temperature range of 551–1000 °C due to the degradation of the framework and the loss of organic components. A similar trend was observed for the Li-NDC MOF, in which the framework is stable up to 554 °C and completely decomposes by 713 °C, resulting in a char weight of 18% (Figure S3b). The peak degradation values obtained for Li-BDC and Li-NDC MOFs are closer to the originally reported values.^29,30^ The major weight loss for the Li-BPDC MOF begins at 496 °C with a weight loss of 71%, demonstrating the framework stability up to 496 °C (Figure S3c). An overall weight loss of 78% occurs in the temperature range of 520–1000 °C, yielding 19% of total weight of the inorganic oxide, Li_2_O. Overall, all three Li-MOFs are thermally stable at higher temperatures compared to most of other transition metal-centered MOFs.^30^ The high thermal stability of Li-MOFs could be due to the lack of solvent molecules incorporated into the crystal structure.^31^ The framework degradation temperatures obtained for all three Li-MOF analogues were aligned with the originally reported values.^29−31^ The thermal stabilities of these isoreticular Li-MOFs were also higher than the thermal stabilities of Li-AOIA and Li-TMCA MOFs.^14^

### Isoreticular Framework Structure and Topology of Li-MOFs

**Figure 3:**
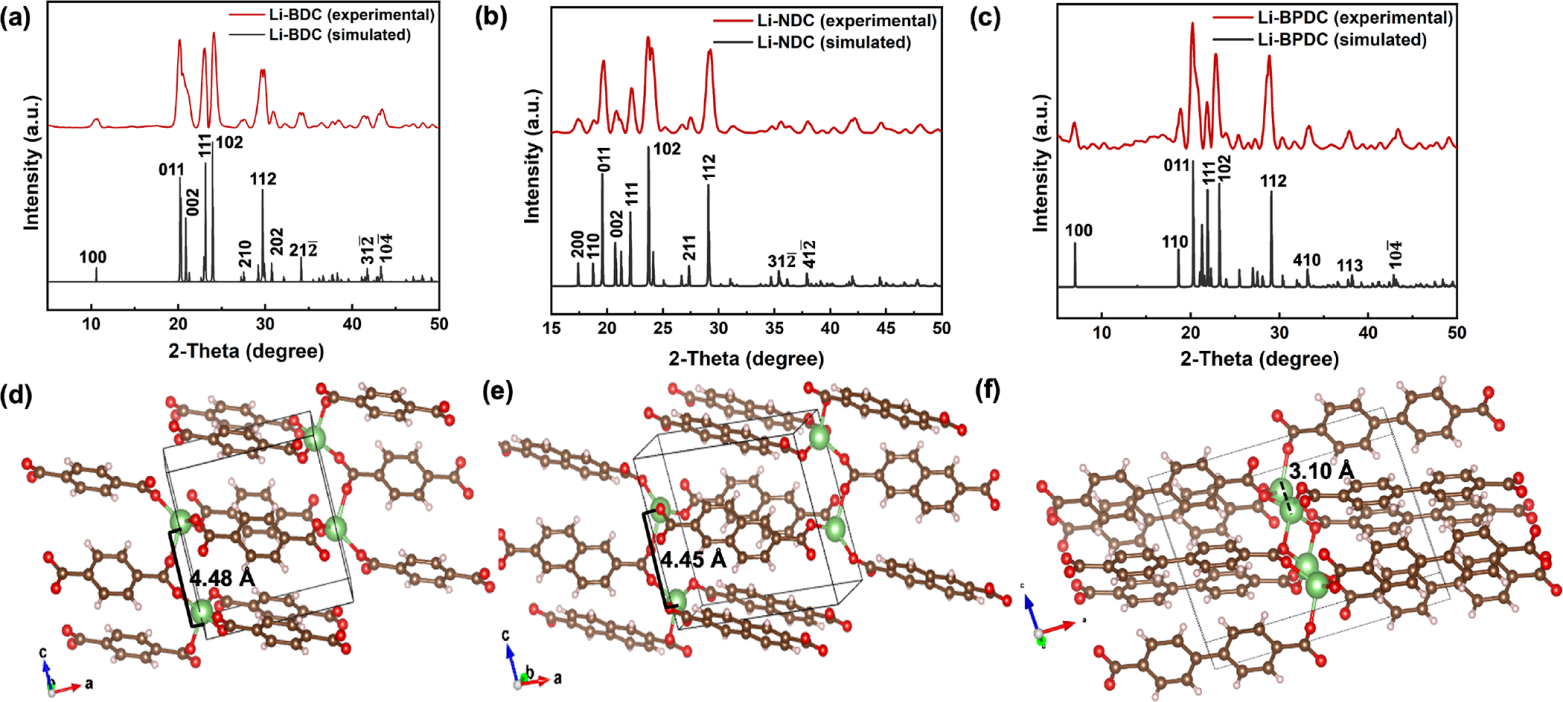
(a–c) Experimental powder XRD traces of isoreticular Li-MOFs along with their respective simulated XRD patterns acquired from the original crystal structures of Li-BDC,^29^ ULMOF-1,^30^ and ULMOF-2;^31^ single crystal structures of: (d) Li-BDC, (e) Li-NDC (ULMOF-1), and (f) Li-BPDC (ULMOF-2), acquired from Cambridge Crystallographic Data Center (CCDC) and Crystallography Open Database (COD) (CCDC 664607, COD ID 4509412, and COD ID 4509710).

The experimental powder XRD traces collected for all three isoreticular MOFs were matched with their respective simulated powder XRDs, acquired from their original crystal structures, reported previously and are depicted in Figure 3a–c.^29−31^ The crystal structures of all three Li-MOFs follow the *P*2_1_/*c* space group, which belongs to monoclinic crystal system (Figure 3d–f). The packing patterns of Li-MOFs follow a 2D-layered arrangement, in which LiO_4_ metal oxide nodes form antifluorite type motifs, consisting of edge-shared tetrahedral nodes.^29−31^ There are no significant changes in the bond distances between neighboring Li metal-ion nodes along the unit cell *c*-axis with respect to the framework expansion in Li-BDC MOF and Li-NDC MOF. The bond distances between two nodes of Li atoms in Li-BDC MOF and Li-NDC MOF are 4.48 and 4.56 Å, respectively, (marked in Figure 3d,e) and aromatic rings are closely packed with face-to-face π–π distances of 5.29 and 5.35 Å, respectively. However, in Li-BPDC MOF, the distance between neighboring two Li^+^ nodes is much closer with a Li–Li bond distance being 3.10 Å (marked in Figure 3f), resulting in rather closely packed face-to-face arrangement of biphenyl units with a π–π distance of 5.13 Å. With the framework expansion, the unit cell length along the *a*-axis increases from Li-BDC to Li-NDC, and Li-BPDC, resulting in gradual increase in the cell volume from 364.89 Å^3^ for Li-BDC, 471.52 Å^3^ for Li-NDC, and 547.34 Å^3^ for Li-BPDC, respectively.

As depicted in Figure 3a–c, the framework’s pore aperture dimensions differ from each other. Li-NDC MOF yields the largest pore aperture width of 5.81 Å, while the length is 11.27 Å. As expected, the pore aperture of Li-BDC is the smallest with the width and length being 4.48 and 9.19 Å, due to the shortest linker length. The Li-BPDC framework structure also yields considerably smaller pore aperture width of 4.46 Å (the smallest pore aperture width) compared to Li-BDC’s and Li-BPDC’s pore aperture widths, due to very closely arranged LiO_4_ nodes but accounted for the largest pore aperture length of 13.52 Å because of the framework expansion.

**Figure 4:**
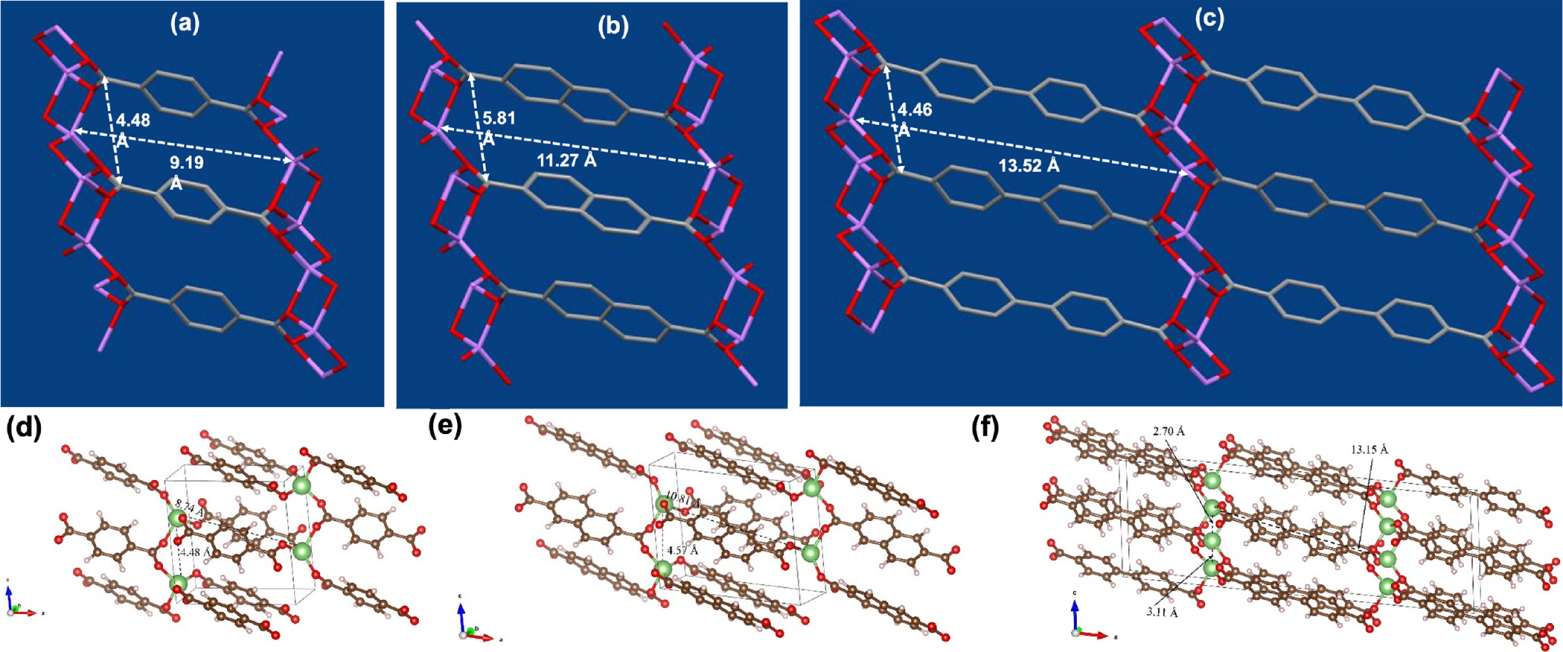
Framework pore aperture dimensions of 1D rectangle voids formed along the *c*-axis of the crystal unit cells of: (a) Li-BDC, (b) Li-NDC, and (c) Li-BPDC; and (d–f) their respective optimized crystal structures with pore aperture dimensions obtained from the computations.

The computational studies conducted for all three Li-MOFs by optimizing their original crystal structures support the pore aperture widths and lengths obtained from their respective original crystal structures by correlating the experimental powder XRD patterns. As shown in Figure 4d–f, for the pristine Li-BDC MOF, computations show that the distances among two neighboring nodes (the pore width and length) of Li atoms are 4.48 and 8.74 Å, which align with the acquired experimental values. For Li-NDC, the computational pore width and length are 4.57 and 10.81 Å, essentially the same as the observed results of 4.56 and 10.82 Å, respectively. The use of BPDC as a longer link further stretches the pore length to 13.15 Å, which is slightly lower than the pore length obtained from the original crystal structure. These changes reflect the gradual expansion of the cell volume from Li-BDC to Li-NDC and Li-BPDC MOFs. Consistent with the experimental finding, in Li-BPDC MOF, the distance between neighboring two Li^+^ nodes are much closer, having a Li–Li bond distance of 3.10 Å, as computations result in a similar value 3.11 Å.

The theoretical porosity volume of each Li-MOF was calculated from the respective unit cell crystal structure using the CrystalMaker X (version 107.2). In general, the porosity (Φ) is defined as the empty volume (*V*_empty_) within a given total volume (*V*_total_) of the unit cell (eq 3).^48^
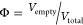
3

The unit cell of Li-BPDC MOF shows the largest porosity volume of 473.89 Å^3^, which accounts for 86.58% porosity per unit cell. The Li-NDC MOF exhibits a porosity volume of 398.32 Å^3^, accounting for 84.47% porosity per unit cell. The Li-BDC MOF resulted in the smallest porosity volume of 292.73 Å^3^, yielding 80.22% porosity per unit cell. However, it is worth noting herein that the accessible porosity (Φ_acc_) is the porosity of interest and depends on the accessible volume for absorbing guest molecules. Thus, identifying the accessible pores/cavities is necessary, as the porosity of interest is usually Φ_acc_. The distribution of accessible pores (and cavities) with respect to the framework expansion also allow us to understand the Li-ion conduction pathways in these porous solid frameworks and the effect of pore size on the feasibility of Li-ion transfer through the porous lattice. Thus, porosity distribution and textural properties of all three MOFs were conducted and are discussed under the textural properties and porosity distribution section.

**Figure 5:**
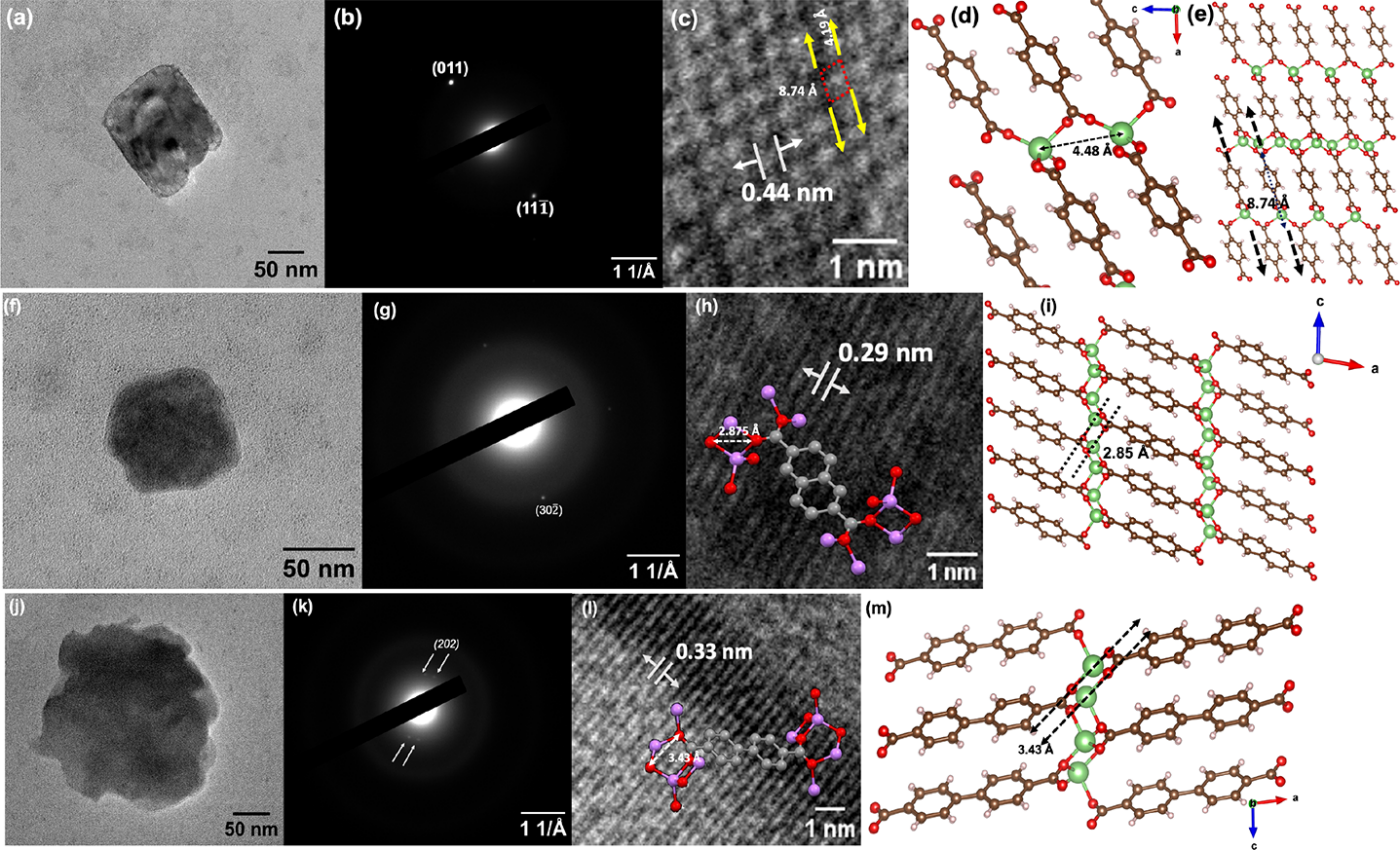
(a) HR-TEM image of a Li-BDC MOF microstructure, (b) its respective SAED pattern, (c) HR-TEM image taken at 300 kx magnification, showing the lattice spacing of d_*(011)*_ of 4.4 Å, which resembles the *d*-spacing between Li^+^ nodes, along with local structure of Li-BDC marked in red color rectangle, (d) respective unit cell crystal structure representing the *d*-spacing between two metal-ion nodes, and (e) supercell lattice of Li-BDC, representing the local structure of the framework, corresponding to the marked red color rectangle in panel c; (f) HR-TEM image of a Li-NDC microstructure, (g) its SAED image, (h) HR-TEM image taken at 300 kx magnification, showing *d*-spacing with respect to the lattice plane of (302̅), in which the *d*-spacing between two oxygen atoms in a tetrahedron node of the asymmetric unit of the crystal structure (shown in the inset), and (i) the respective supercell model marked with the *d*-spacing measured from the crystal lattice (marked in dotted lines); and (j) HR-TEM images of a Li-BPDC microstructure, (k) its SAED image, representing the diffractions from (202) lattice plane, (l) a HR-TEM image taken at 200 kx magnification, along with the measured the *d*-spacing, corresponded to the distance between two neighboring oxygen atoms in the tetrahedron nodes, (m) the respective unit cell structure, with the *d*-spacing between two oxygen atoms in the tetrahedron nodes.

Microstructures of isoreticular Li-MOFs visualized from HR-TEM are shown in Figure 5 and reveal the morphologies of microstructures along with their crystallinity, lattice arrangements, and unit cell parameters of nanocrystals, providing insight into their local framework structures. A microstructure of Li-BDC exhibits a cubic shape morphology with visible voids (micropores) scattered within the microstructure, indicating a porous microstructure (Figure 5a) of self-assembled nanocrystals. The SAED pattern of Li-BDC shown in Figure 5b reveal diffractions from two dominant lattice planes (Figure S4a), corresponding to the Miller indices of (011) and (111̅), having the lattice *d*-spacing of *d*_(011)_ = 4.39 Å and *d*_(111̅)_ = 3.93 Å, respectively. The HR-TEM image of a Li-BDC microstructure (Figure 5c), obtained at 80 kV view along the [010] zone axis, exhibits the local structure of Li-BDC MOF’s unit cell’s metal-ion nodes, with a *d*-spacing of 4.4 Å, which resembles the *d*-spacing between two Li metal-ion nodes along the *c*-axis of the unit cell, as represented in Figure 5d. The local structure of Li-BDC framework was able to configure from the Figure 5c HR-TEM image (marked by red dotted line rectangle) and is resembled to the supercell of Li-BDC shown in Figure 5d, in which edge-to-edge distance from an oxygen atom of a tetrahedron node to a Li-ion of a tetrahedron node along the *a*-axis is measured to be 8.74 Å and agrees with the edge-to-edge length of the marked rectangle. The edge-to-edge width of the rectangle was measured to be 4.19 Å, which resembles the distance between two neighboring metal oxide nodes (O–Li) along the *c*-axis, representing the local framework structure of the Li-BDC unit cell.

The TEM image of a Li-NDC microstructure shown in Figure 5f exhibits slightly truncated cubic shape morphology with a rather dense structure compared to Li-BDC microstructures with visible voids. The SAED pattern of Li-NDC (Figure 5g) yielded a poorly resolved lattice plane, which corresponds to the (302̅) plane (Figure S4b). The lattice *d*-spacing of *d*_(302̅*)*_ = 2.87 Å that corresponds to the distance between two oxygen atoms in a tetrahedron node of the asymmetric unit of the crystal structure (shown in the inset of Figure 5h) well agrees with the *d*-spacing measured from the HR-TEM image of Figure 5h. The corresponding supercell structural model view along the *b*-axis with the respective lattice *d*-spacing (marked in two dotted lines) is depicted in Figure 5i and confirms that self-assembled nanocrystals follow the originally reported crystal structure of ULMOF-1. A microstructure of Li-BPDC visualized under HR-TEM reveals an irregular shape morphology with a few visible voids (in lighter see through spots), as shown in Figure 5j. The SAED pattern (Figure 5k) exhibits a weak diffraction pattern, which belong to the lattice plane of *(202)*, having the *d*-spacing of *d*_*(202)*_ = 3.30 Å (Figure S4c). The lattice *d*-spacing measured from the HR-TEM image of Figure 5l closely agrees with the *d*-spacing between two neighboring oxygen atoms in a tetrahedron node of an asymmetric unit (inset of Figure 5l) and is represented from the crystal structure model in Figure 5m.

### Textural Properties and Porosity Distribution

To reveal the Li-ion conductivity in these solid porous materials, it is necessary to understand their textural properties and porosity distributions. The variation in pore volume and pore area with respect to the framework structure in Li-MOFs could benefit transporting mobile Li-ions via porous channels. As this is the first report on the textural properties and porosity distribution of isoreticular Li-MOFs, we have conducted an in-depth study on the adsorption–desorption behavior of Li-MOFs’ microstructures by acquiring full N_2_-adsorption–desorption isotherms for powder samples of Li-MOFs at 77 K. The N_2_-adsorption Brunauer–Emmett–Teller Surface area (*S*_BET_) and the BJH porosity distribution of microstructures were obtained from Brunauer–Emmett–Teller analyses and Barret-Joyner-Halenda (BJH) analyses, respectively, and are summarized in Table 1. The adsorption data were also analyzed using the nonlocal density functional theory (NLDFT) approach that allows quantification of both accessible micro- and mesopores. The cumulative pore area and pore volume deduced from the NLDFT method are also included in Table 1. All three Li-MOFs exhibit considerably larger BET surface area (*S*_BET_), and the microstructures of Li-NDC yield the largest surface area (*S*_BET_), resulting in the highest BJH desorption cumulative pore volume and pore area compared to the microstructures of Li-BDC and Li-BPDC. However, it is normally stated that, in some instances, the BET method is not suitable for microporous materials, especially for MOFs.^49−51^ BET surface area can overestimate for MOFs with micropore distributions (pore width <20 Å) because of the enhanced physical adsorption by micropore filling.^49−51^ On the other hand, it is suggested that the BET analysis is applicable for microporous solids if the micropore width is more than the bilayer thickness of nitrogen (7 A°). Thus, selecting the appropriate adsorption data in the pressure range corresponding to monolayer completion^52^ is necessary to correct the overestimated surface area by BET. Therefore, herein, we also report the single-point surface area at the relative pressure *P*/*P*_0_ of 0.3 and surface areas were found to be 410.49, 446.10, and 382.63 m^2^/g for Li-BDC, Li-NDC, and Li-BPDC, respectively. In both analyses, Li-NDC yielded the largest surface area compared to Li-BDC and Li-BPDC MOFs. Regardless the framework expansion in Li-BPDC MOF, the largest surface area in Li-NDC MOF clearly agrees with the packing arrangement of organic ligands, yielding in a wider framework pore aperture (Figure 4b) compared to Li-BDC’s and Li-BPDC’s framework pore apertures thereby having the largest cumulative pore volume and pore area. The smallest surface area, BJH desorption cumulative pore volume, and pore area obtained for Li-BPDC speak to its closely packed biphenyl units in the framework structure with a considerably narrow framework pore aperture width (Figure 4c)**.**

**Table 1:** Results of BET, BJH, and NLDFT Analyses for the Microstructures of Li-MOFs

Li-MOFs	*S*_BET_ (m^2^/g)	BJH desorption cumulative pore volume (cm^3^/g)	BJH desorption cumulative pore area (m^2^/g)	cumulative pore area by NLDFT (m^2^/g)	cumulative pore volume by NLDFT (cm^3^/g)
Li-BDC	834.84 ± 15.58	0.650	569.23	437.84	0.695
Li-NDC	894.76 ± 9.78	0.708	614.46	465.87	0.737
Li-BPDC	741.53 ± 9.78	0.618	526.61	407.16	0.648

The porosity distribution calculated from the NLDFT approach also exhibits a similar trend where Li-NDC possesses the highest cumulative pore volume and pore area compared to the other two Li-MOFs, supporting the BJH analyses (Table 1). However, the cumulative pore areas obtained for each Li-MOF from NLDFT approach was slightly lower than the corresponding BJH desorption cumulative pore area. On the other hand, the cumulative pore volumes deduced for each Li-MOF from NLDFT approach closely agree with the BJH analyses, confirming that the high-density pore distribution is in the micropore and narrow mesopore regions. The larger mesopores could have resulted from the void spaces among the self-assembled nanocrystals rather than from the actual cavities within the MOF framework.

The corresponding full N_2_-BJH adsorption–desorption isotherms, obtained after subjected adsorption–desorption data to the Chi-square goodness of fit test, are shown in Figure S5a. All three Li-MOFs exhibit Type I(b) reversible isotherms, revealing porous structures with the presence of both micropores (≤2 nm) and narrow mesopores (≤15 nm).^53^ The lack of hysteresis in adsorption and desorption isotherms confirms that the adsorption and desorption processes are fully reversible. This further confirms that pores are cylindrical in geometry with no pore blocking or percolation effect, which occurs if the pore has a narrow neck as in ink-bottleneck.^54^ The BJH adsorption–desorption d*V*/d*w* pore volumes with respect to Li-MOFs’ pore widths, depicted in Figure S5b, revealed that microstructures of Li-MOFs exhibit bimodal porosity distributions, in which largely in the wider micropore area (18 Å ≤ 25 Å), and mesoporous area with median pore width in the range of 25–100 Å and larger mesopores in the range of 100–250 Å. The average BJH adsorption pore widths for Li-BDC, Li-NDC, and Li-BPDC were 45.97, 45.27, and 47.26 Å, respectively, and reclined to narrow mesopore size range (4.5–4.7 nm).

To get the accurate average pore width in the micropore region, we also applied the Horvath–Kawazoe semiempirical model, which specifically applies to determine the pore size distribution in microporous materials.^55^ The micropore width distribution ranges from 7 to 25 Å for all three Li-MOFs and the average micropore widths are found to be 18.71 18.65, and 18.59 Å for Li-BDC, Li-NDC, and Li-BPDC, respectively. The average micropore width in Li-BPDC is slightly narrower than the average micropore widths of Li-NDC and Li-BDC, providing perhaps an optimal micropore volume distribution for guest molecules to occupy.

**Figure 6:**
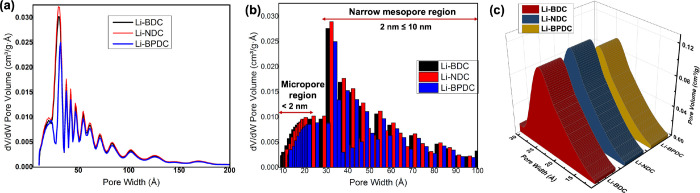
Porosity distribution plots obtained from NLDFT analysis for Li-MOFs: (a) line graph of d*V*/d*w* pore volume distribution, (b) bar graph of d*V*/d*w* pore volume distribution, and (c) 3D plot of pore volume distribution in the micropore size region.

The adsorption isotherms analyzed by the NLDFT approach have yielded rather accurate pore volume distributions in both microporous (10–20 Å) and mesoporous range (20–200 Å) for all three Li-MOFs (Figure 6a). The cumulative bar graph of d*V*/d*w* pore volumes with respect to pore width shown in Figure 6b clearly indicates that the microstructures of Li-NDC show the largest d*V*/d*w* pore volume distribution in both microporous and mesoporous regions, whereas the microstructures of Li-BPDC possess the smallest bimodal d*V*/d*w* pore volume distribution. The considerably narrow micropore volume distribution in Li-BPDC compared to the micropore volume distributions in both Li-NDC and Li-BDC clearly agrees with Li-BPDC’s crystal lattice packing pattern, having a closely packed biphenyl units in the framework. The high-density bimodal (microporous and mesoporous) porosity distribution (Figure 6b) observed in all three microstructures further explains the considerably high surface area (*S*_BET_), like the most isoreticular MOFs, which belong to prototypical MOF-5 generation.^3,11^ The 3D plot of pore volume distribution in the micropore size range (10–30 Å)for Li-MOF microstructures shown in Figure 6c confirms that the cumulative micropore volume decreases in a manner of Li-NDC > Li-BDC > Li-BPDC, although the isoreticular framework expands with respect to the ligand length along the vertices of the framework from Li-BDC to Li-NDC to Li-BPDC, respectively. Thus, overall, the isoreticularity in Li-MOFs clearly signifies the effect on their optimal pore aperture.

### Li-Ion Conduction in Isoreticular Li-MOFs

MOFs with open metalation concept (OMC) can easily participate in Li-ion conduction via either successive doping of lithium salts to interact with open metal sites (OMSs) or by post synthetic grafting onto the activated OMSs via modification of secondary building units (SBUs). Such post synthetic modifications allow furnishing an adequate amount of mobile Li^+^ ions to achieve reasonable room-temperature ionic conductivity of the order of 10^–5^ S/cm.^14^ However, in our case, as evidenced from the XPS and XRD analysis, none of the isoreticular Li-MOFs has solvent molecules coordinated to the tetrahedron Li-ion metal centers, where unsaturated OMSs can readily generate by removing the coordinated solvent molecules via inert thermal activation processes. Thus, our thesis hypothesis for the Li^+^ conduction may follow a pore filling mechanism of lithium salts via occupying mobile Li-ions in accessible pores of the framework.^56,57^ Successive pore filling of lithium salts (LEC) into the framework’s cavities may allow us to verify and establish the Li^+^ conduction in isoreticular Li-MOFs. The differences in ionic conductivities could reveal the subtle effect of their isoreticular framework expansion, thereby varying the pore filling capacity and binding with the framework for the mobile Li-ions. Herein, our intention is to study the Li-ion conduction, reveal its mechanism, and correlate the isoreticularity with the ionic conductivity.

To elucidate our thesis hypothesis on Li-ion conduction via a pore filling mechanism, the electrochemical impedance spectra (EIS) of solid pellets of LEC@Li-MOFs were acquired at different temperatures, ranging from 25 to 55 °C. To rule out the interference of proton conductivity with the Li^+^ conduction, due to any trace amount of moisture present in MOFs, powder samples of MOFs were heated at 250 °C in a vacuum oven prior to make electrolytes. Additionally, the powder samples of electrolytes collected from the LEC solution was dried over 72 h in a vacuum at room temperature to remove any traces of moisture prior to make the pellets. Thus, the pellets were dry solid pellets at the temperature range from 25 to 55 °C. The XPS elemental composition analysis confirms the retention of LiClO_4_ and EC in the pellets from the elemental percent weight increased in Li and oxygen content (Table S2). The binding energy spectra of LEC@Li-MOFs for C 1s, O 1s, and Li 1s show the incorporation of EC and LiClO_4_ from the additional binding energy peaks (Figure S6). The presence of additional binding energy peaks at 286.1 and 290.8 eV in the C 1s spectra confirms the incorporation of EC along with the binding energy peaks at 533.4 eV for ether oxygen in O 1s spectra. Appearance of an additional shoulder peak at 57.9 eV in the Li 1s spectra confirms the retention of LiClO_4_.

**Figure 7:**
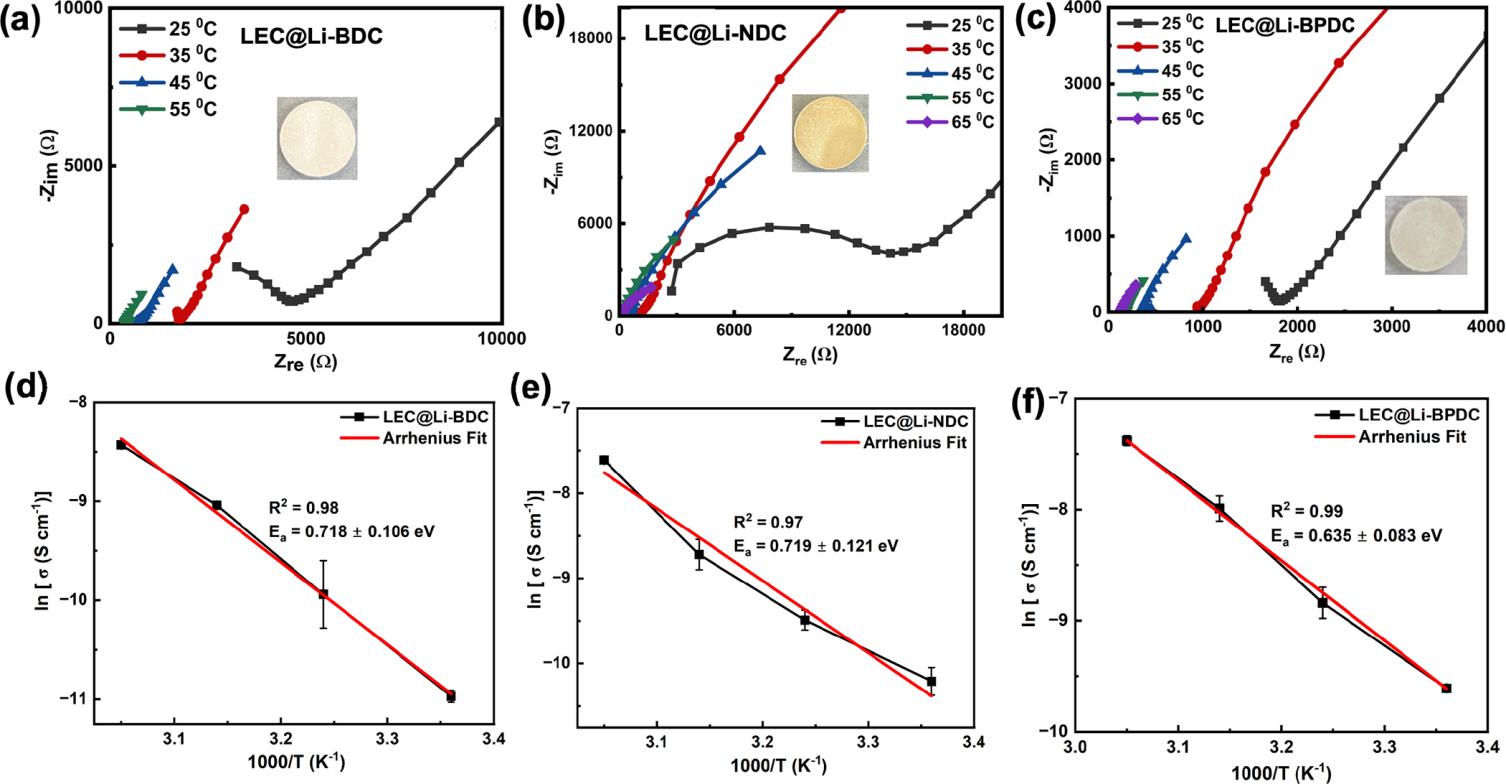
(a–c) Temperature-dependent electrochemical impedance spectra obtained after loaded Li-MOFs with LEC to deduce the temperature-dependent Li-ion conductivities, represented in Nyquist plots, and (d–f) respective Arrhenius plots for LEC@Li-BDC, LEC@Li-NDC, and LEC@Li-BPDC (error bars are marked with black lines).

The respective Nyquist plots used to deduce the temperature-dependent ionic conductivities are represented in Figure 7a–c. The pellets prepared from pristine MOFs show open circuit current (Figure S7), ruling out the proton conduction, and further confirm that the powder samples are free of moisture. At room temperature, the conductivities of LEC@Li-BDC, LEC@Li-NDC, and LEC@Li-BPDC were found to be 1.72 × 10^–5^, 3.66 × 10^–5^, and 6.66 × 10^–5^ S cm^–1^, respectively. The ionic conductivities of LEC@Li-MOFs are 1–2 orders of magnitude higher than the previously published Li-MOFs derivatives, in which open metal sites were doped with anions.^14^ However, all three LEC@Li-MOFs’ conductivity values are approximately an order of magnitude below compared to the Li-ion conduction in the state-of-the-art functional polymer-based solid-state battery electrolytes at room temperature.^58^ With the isoreticular expansion of the framework, we observed gradual increase in the ionic conductivities, approximately by 4-fold and 2-fold in LEC@Li-BPDC, compared to the ionic conductivities in Li-BDC@LEC and Li-NDC@LEC, respectively. As the diffusion kinetics of guest molecules largely depends on the pore size,^8,9^ high-density-interconnected accessible pores with different pore volume distributions can occupy Li-ions, EC, and ClO_4_^–^ ions in different capacities, yielding either high ionic conductivity or low ionic conductivity. In our case, the Li-BPDC MOF holds the smallest cumulative pore volume (Table 1 and Figure 6) but yielded the highest ionic conductivity. In contrast, the Li-NDC MOF and Li-BDC MOF possess considerably larger pore volumes (Figure 6 and Table 1), but the ionic conductivities are lower compared to the ionic conductivity of LEC@Li-BPDC, suggesting that pore volume plays a role in Li^+^ conduction. It is also possible to rationalize that larger pores can create distance barrier to move faster for mobile Li-ions between pores. Abetting our findings, a past research study has investigated the effect of porosity on Li-ion conductivity in a tetraarylborate polymer network and has found that increasing the porosity led to significant decreases in ionic conductivity due to the large distance barrier between adjacent hopping sites, resulting in high activation energy. The prior research has also shown that MOFs with smaller pore volume exhibit higher proton conductivity by 2–3 orders of magnitude because of a smaller pore volume minimizes the number of water molecules in the pores, in turn minimizing the number of hydrogen bonds.^59^ However, an opposite trend has been observed for Mg^2+^ conduction in MOFs with larger pores (e.g., framework expanded MOF-74), resulting in significantly faster transport of Mg-ions than does its parent MOF with smaller pores.^60^

As our findings suggest that there could be an optimal pore volume for the subtle effect on Li-ion conduction in these porous solids, it is crucial to understand the Li-ion diffusion by correlating it with the thermal activation process of the Li-ion conduction in LEC@Li-MOFs. Thus, we acquired temperature-dependent ionic conductivities of LEC@Li-MOFs solid pellets and studied the impact of the isoreticularity of Li-MOFs’ on the activation energy for Li^+^ conduction below the glass transition temperature for each solid electrolyte. Table 2 summarizes the temperature-dependent ionic conductivities and corresponding activation energies calculated for LEC@Li-MOFs, by applying the Arrhenius equation (eq 2). At higher temperatures (above room temperature), we observed gradual increased in the ionic conductivities where all three LEC@Li-MOFs exhibit high ionic conductivities with an order of magnitude increment at 45 and 55 °C. However, above 55 °C the thermal stability of pellets in the solid form was found to be unstable, and beyond this temperature, pellets became soft due to slow melting of EC, thereby yielding somewhat higher ionic conductivities (Figure S8), comparable to MOF-based quasi-solid electrolytes.^18,61^ Additionally, the TGA plots of LEC@Li-MOFs (Figure S9) exhibit sharp weight losses at 106, 112, and 126 °C, respectively, evidencing the melting followed by degradation of EC.

**Table 2:** Summary of Temperature-Dependent Ionic Conductivities Obtained from Electrochemical Impedance Measurements and Respective Activation Energies Calculated for LEC@Li-MOFs

	temperature-dependent ionic conductivities (σ_(T)_, S cm^–1^)				
LEC@MOFs	25 °C (x10^–5^)	35 °C	45 °C (x10^–4^)	55 °C (x10^–4^)	activation energy (eV)
Li-BDC	1.72 ± 0.10	4.80 ± 1.60 × 10^–5^	1.18 ± 0.01	2.17 ± 0.04	0.719 ± 0.106
Li-NDC	3.66 ± 0.62	7.49 ± 0.87 x10^–5^	1.63 ± 0.31	4.91 ± 0.29	0.718 ± 0.121
Li-BPDC	6.66 ± 0.24	1.44 ± 0.20 x10^–4^	3.36 ± 0.37	6.19 ± 0.26	0.635 ± 0.083

In general, the activation energy simply describes the conductivity of the thermally activated ion transport process in an amorphous phase.^62^ The experimental and linear fitted Arrhenius plots of ln σ_(T)_ against the reciprocal temperature from 25 to 55 °C are shown in Figure 7d–f, which follow somewhat linear correlation for the temperature dependence of the conductivities in all three electrolytes with linear correlation coefficient (*R*^2^) values of 0.98, 0.97, and 0.99 for LEC@Li-BDC, LEC@Li-NDC, and LEC@Li-BPDC, respectively. However, in comparison to a rather linear Arrhenius plot with the highest linear correlation coefficient of LEC@Li-BPDC, the other two Li-MOFs’ electrolytes yielded poor fits (lower *R*^2^ value) for the Arrhenius relation, deviating from a linear representation at 35 °C, implying an unfavorable thermally activated ion transport process. Activation energies (*E*_a_) of pellet electrolytes were calculated from the slopes of the linear fitted Arrhenius plots and are summarized in Table 2. Since the activation energies of all three electrolytes are within the margin of error bar, it is difficult to state a comparable conclusion about the thermally activated ion transport process. Overall, the activation energies in all three solid electrolytes are considerably higher compared to the most reported MOF-based solid and semisolid Li-ion conductors,^20,21^ including the recently reported Li-MOF-based solid-state electrolytes (Li-AOIA@BF_4_).^14^ However, in these MOF-based solid-state electrolytes, Li-ion conduction was facilitated by grafting lithium salts to OMSs, allowing the Li^+^ to move relatively freely in the framework channels.^14,20^ In our LEC@Li-MOF electrolytes, we rationalize that considerably high activation energies resulted because the Li^+^ conduction mechanism is different from the solid-state electrolytes constructed from lithium salt-grafted MOFs. We hypothesize that the Li-ions move through the porous channels via a pore filling mechanism, which may follow the diffusion of Li-ions from pore to pore by interacting with EC and the functional sites of the framework, and then move to the neighboring unit cell of the microstructure via either interconnected porous channels or hopping through the framework. Thus, the activation energy obtained from the Arrhenius plots for our solid electrolytes could be the total energy, which takes Li^+^ to diffuse through the pore channels and move to the neighboring unit cell in the Li-MOF microstructures.

Based on the BET results, as accessible pore volume decreases in the manner of Li-NDC > Li-BDC > Li-BPDC, the activation energy needed for Li-ion diffusion through the smaller porous channels is lower compared to the crystalline solid lattice with larger porous channels. This is because the diffusion distance for Li-ions is less in smaller pores compared to the diffusion distance in larger pores, consuming higher energy to overcome the diffusion barrier between pores. Additionally, the bimodal pore size distribution (Figure 6b) in all three Li-MOFs could also play a role in diffusion of Li^+^ ions from one pore to the other, as smaller pores increase the confining effect compared to larger pores. Moreover, densely packed vs loosely packed pore distribution as well as the excess cavities may increase the hopping distance between pores, approximating a bulk electrolyte behavior within the pores.^63^ As we observed from the porosity distribution data, Li-BPDC MOF’s narrower micropore and mesopore distribution provides perhaps a suitable pore volume distribution for Li^+^ diffusion, favoring an energetically facile Li-ion mobility in Li-BPDC MOF. However, we cannot rule out the morphological differences contributing from crystallite size and pore alignment of each Li-MOFs. These factors could also play a subtle effect on the Li^+^ diffusion, contributing to the grain boundaries effect for Li^+^ conduction.^64,65^ Although we have not conducted in-depth studies on the effect of grain boundaries in our current study, we make an effort to understand the effect of crystallite size of each MOF on the Li^+^ diffusion and correlate to the Li^+^ conductivity. For each Li-MOF, we calculated the crystallite sizes according to the Scherrer formula (eq 4) for the most intense diffraction peaks at the Miller indices (hkl) of (011), (111), and (102).
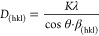
4where *K* is the particle shape factor (0.9), λ is the X-ray wavelength, β_hkl_ is the half-width of (hkl) reflection, and θ = 2θ/2 is the Bragg angle corresponding to (hkl) reflection.

The average crystallite sizes were calculated to be 12.45, 14.36, and 11.78 nm for Li-MOFs of Li-BDC, Li-NDC, and Li-BPDC, respectively. The crystallite sizes decrease in the manner of Li-NDC > Li-BDC > Li-BPDC, following the similar trend as their respective pore aperture volume. In crystalline and polycrystalline materials, the past research has evidenced that grain size correlates to the ionic conductivity. In crystalline materials, ionic conductivity increases gradually with respect to the grain size but becomes static after reaching to a certain grain size.^64^ In our case, we observe the opposite trend where ionic conductivity increases with the decrease in crystallite size, suggesting that perhaps there is a cooperative effect of the crystallite size and the pore volume on the differences in ionic conductivities. Nonetheless, in-depth understanding on the mobile Li^+^ interaction with the framework functional sites and the EC matrix is necessary for a definitive confirmation as the pore volume and binding interactions play a cooperative role along with the grain size of Li-MOFs for ion conduction. Thus, we combined experimental results with computational analysis for deducing the Li^+^ interactions with the EC and the Li-MOFs’ framework and elucidated their Li^+^ ion conduction mechanisms.

### Li-Ion Conduction Pathway

**Figure 8:**
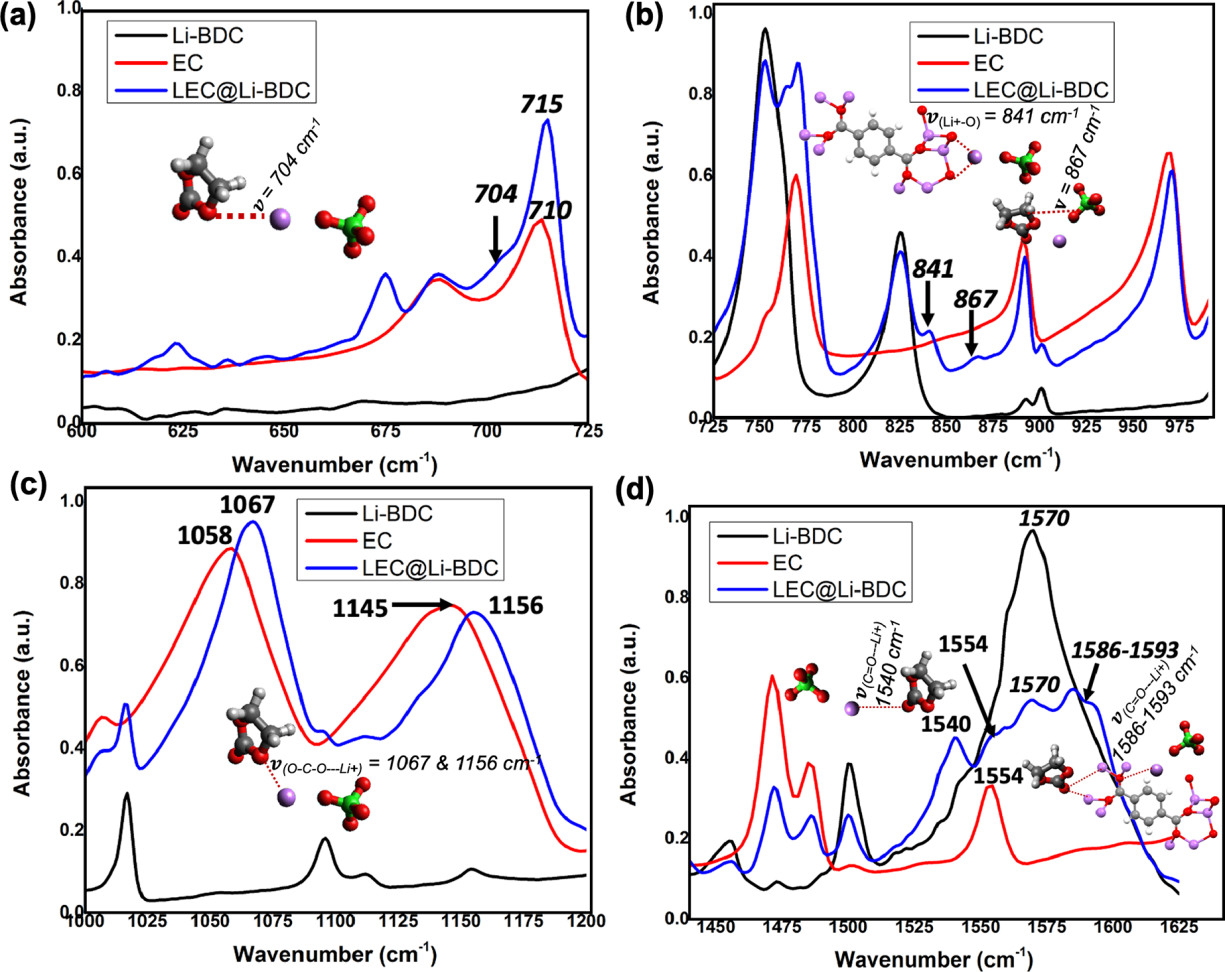
Normalized FTIR spectra of LEC@Li-BDC, Li-BDC, and EC for the selected frequency region of: (a) 600–725 cm^–1^: inset—the chemical bonding interactions of Li^+^ with the EC structure, and counteranion ClO_4_^–^; (b) 725–990 cm^–1^: inset—the chemical bonding interaction of Li^+^ with an oxygen atom of a Li_2_O metal node in an asymmetric unit of Li-BDC, and the solvation interactions between EC and ClO_4_^–^; (c) 1000–1200 cm^–1^: inset—the chemical bonding interactions of Li^+^ with ether oxygen in the EC structure and counteranion ClO_4_^–^; and (d) 1450–1625 cm^–1^: inset—the chemical bonding interactions of Li^+^ with carbonyl oxygen in the EC structure and EC’s carbonyl group with Li^+^ metal nodes in an asymmetric unit of Li-BDC.

**Figure 9:**
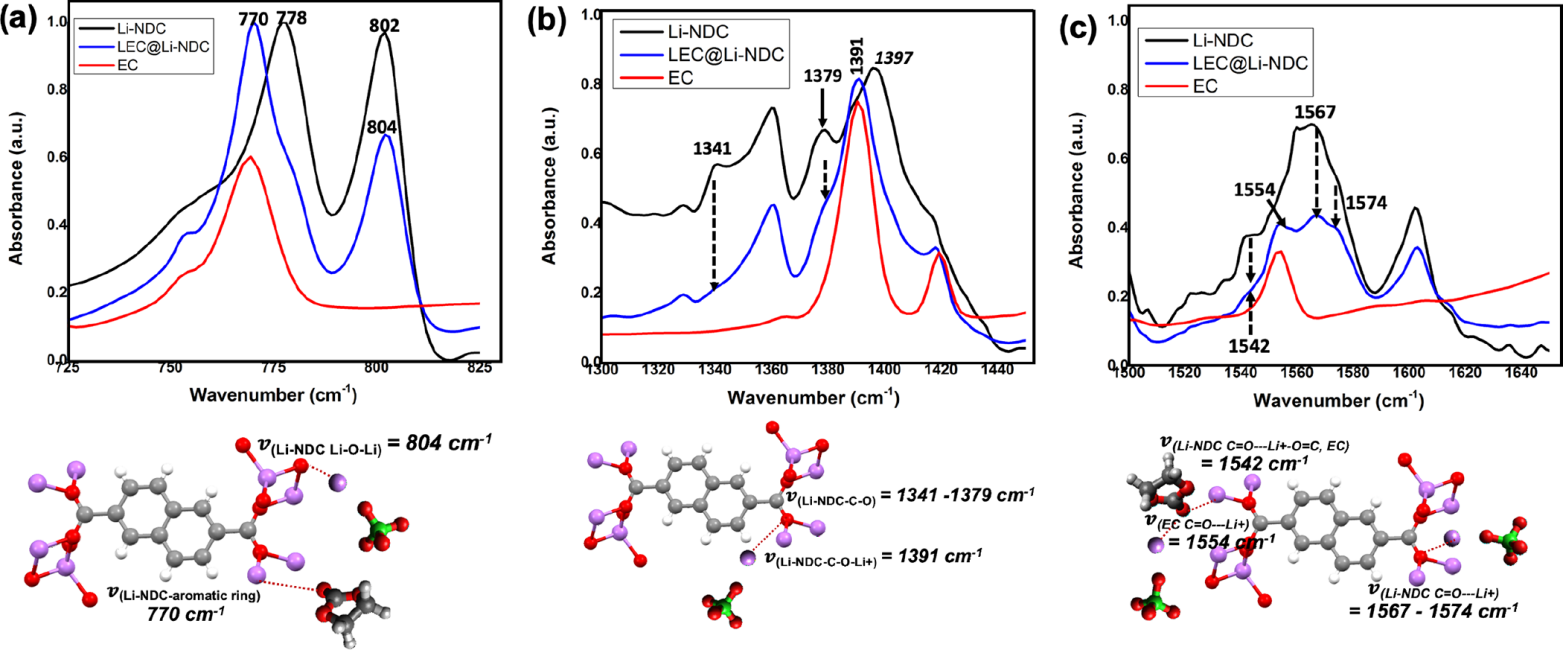
Normalized FTIR spectra of LEC@Li-NDC MOF, Li-NDC MOF microstructures, and EC for the selected frequency regions of: (a) 725–825 cm^–1^, (b) 1300–1450 cm^–1^, (c) 1500–1650 cm^–1^, along with structural representation of binding interactions and vibronic stretching modes of functional groups in Li-NDC asymmetric unit, EC, and LiClO_4_.

**Figure 10:**
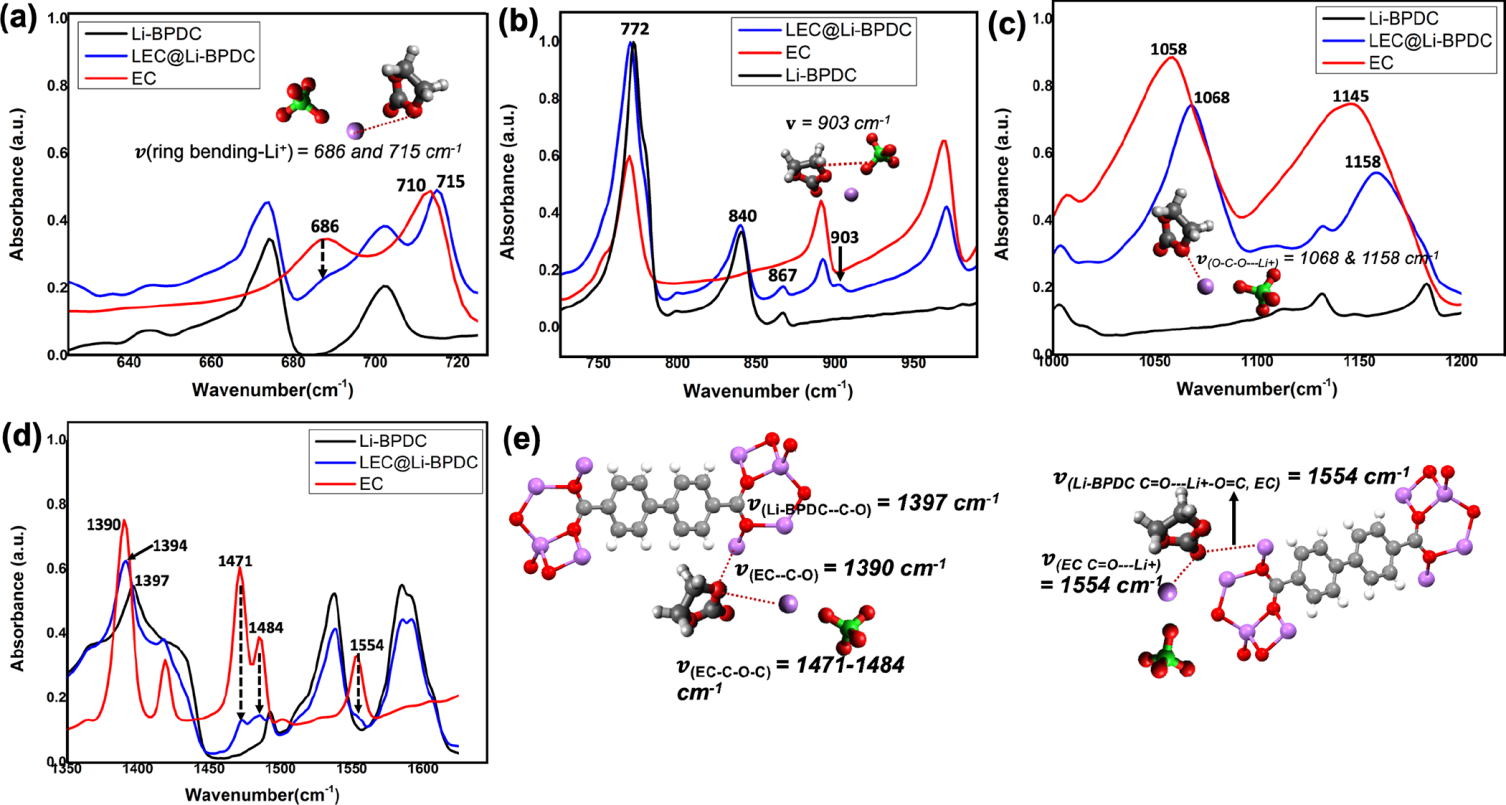
Normalized FTIR spectra of LEC@Li-BPDC, Li-BPDC microstructures, and EC for the selected frequency regions of: (a) 625–725 cm^–1^, (b) 725–990 cm^–1^, and (c) 100–1250 cm^–1^: and (c) 1350–1625 cm^–1^, along with structural representation of binding interactions and vibronic stretching modes of functional groups in the Li-BPDC asymmetric unit, EC, and LiClO_4_.

We investigated binding interactions of Li-MOF microstructures with the lithium salt and plasticizer (EC), by acquiring FTIR spectral traces for LEC@Li-MOF pellets and pristine powder samples of Li-MOF microstructures and EC. The FTIR spectra obtained are depicted in Figures 8–10,along with the structural representation on the bonding modes of the functional group interactions in Li-MOFs, EC, and LiClO_4_, and their respective vibronic stretching frequencies (ν). Figure 8 shows the FTIR absorption spectra for LEC@Li-BDC MOF, pristine Li-BDC MOF microstructures, and EC for the frequency regions, corresponding to EC’s ring bending mode (Figure 8a, Li-BDC MOF’s aromatic ring bending modes (725–780 cm^–1^) and Li–O metal oxide node stretching modes (800–850 cm^–1^), along with EC’s ring breathing modes (850–975 cm^–1^, Figure 8b), EC’s O–C–O stretching modes (Figure 8c), and Li-BDC MOF’s and EC’s carbonyl stretching modes (Figure 8d).

The binding interaction of Li^+^ ions in LiClO_4_ with EC due to the solvation can usually be located from the changes in EC’s ring bending and ring breathing modes at the vibronic frequency of 710 and 890 cm^–1^.^66^ In the FTIR absorption spectrum of LEC@Li-BDC, the EC’s ring bending and ring breathing modes exhibit recognizable changes. Appearing weak shoulder bands at 704 and 867 cm^–1^ (Figure 8a,b) confirm the binding of Li^+^ and ClO_4_^–^ with EC’s ring oxygen and carbonyl group, respectively.^66^ The shift in the EC’s ring bending mode band at 710 cm^–1^ to the high vibronic frequency at 715 cm^–1^ further supports the EC’s interactions with LiClO_4_, confirming the formation of the solvation shell of Li^+^. The reduction in intensity of the benzene ring bending peak at 753 cm^–1^ along with the slight shoulder peak at 764 cm^–1^ provides additional evidence for the binding interactions of solvated Li^+^ ions with the oxygen atoms in the metal oxide nodes of the frameworks (Figure 8b).

Overall, the absorption frequencies elucidated to identify the potential binding interactions of mobile Li-ions with the Li-BDC MOF’s functional framework structure have yielded comprehensive insight into how EC and LiClO_4_ participate in Li^+^ conduction. These binding interactions with functional groups of the asymmetric units in the Li-BDC MOF and carbonate functionality in EC, which provides the strong solvation interactions with Li^+^ in LiClO_4_, appears to be catalyzing the dissociation of LiClO_4_ into mobile Li^+^ and ClO_4_^–^. Then, the dissociated Li-ions move through the porous channels, interacting with EC’s carbonate functional sites, the framework’s metal oxide nodes, and organic linker’s carbonyl groups. The significant attributes observed in the vibronic frequencies evidence these highly cooperative binding interactions, involved in both EC and the Li-BDC framework’s functional sites with mobile Li-ions.

Additionally, the vibronic shifts in the main peaks of EC centered at 1058 cm^–1^ and its secondary band at 1145 cm^–1^, which are assigned to (C–O–C) stretching mode and its combination mode, respectively, to the higher frequencies at 1067 and 1156 cm^–1^, confirm the mobile Li^+^ interactions with the ether oxygen of EC (Figure 8c).^67^ The binding interactions of mobile Li^+^ with the Li-BDC framework’s functional groups are able to identify from the associated changes in the vibronic stretching bands of metal oxide nodes at ν_(Li–O)_ 826 cm^–1^ in Figure 8b and the carbonyl stretching modes at ν_(C=O)_ 1570 cm^–1^ and ν_(C=O–Li)_ 1554 cm^–1^ in the frequency region from 1540 to 1593 cm^–1^ in Figure 8d. The interactions of mobile Li^+^ with the oxygen atoms in the Li-BDC MOF’s metal oxide nodes is evidenced by the appearing of an additional shoulder peak at the vibronic frequency of 841 cm^–1^, with a slight intensity reduction of original stretching band at ν_(Li–O)_ = 826 cm^–1^ (Figure 8b). The intensity reduction along with the peak broadening observed for the ν_(C=O–Li+)_ at 1570 cm^–1^, shown in Figure 8d, associates to the formation of additional shoulder peaks at 1586 and 1593 cm^–1^. These attributes are assigned to the binding interactions of C=O–Li^+^ nodes with EC’s carbonyl oxygen. The EC’s CH_2_ bending vibrations,^68,69^ centered at 1554 cm^–1^ in pure EC, exhibits reduced absorption in LEC@Li-BDC, featuring a weak shoulder, which is associated to the binding of the carbonyl oxygen with Li^+^ in the framework’s metal oxide nodes. A secondary band at the lower frequency of 1540 cm^–1^ to the pure EC’s CH_2_ bending mode vibration at 1554 cm^–1^ evidences additional binding interactions of EC’s carbonyl oxygen with solvated Li-ions (Figure 8d).

In contrast, the FTIR absorption spectra obtained for LEC@Li-NDC along with pristine powder samples of Li-NDC microstructures and EC exhibit some noticeable differences in binding interactions with the lithium salt (Figure 9 and Figure S10). We observe no associated broadening developed besides the EC’s ring bonding mode band at 710 cm^–1^, indicating very weak binding interactions between Li^+^ in LiClO_4_ and EC from the solvation effect (Figure S10a). The absence of additional attributes or peak broadening to the EC’s breathing mode band at 890 cm^–1^ (Figure S10b) and EC’s central stretching modes of C–O–C at 1058 and 1145 cm^–1^ proves that there are no noticeable bindings between EC and ClO_4_^–^ and between EC and Li^+^, respectively. Interestingly, in terms of interacting lithium salt and EC with the Li-NDC framework’s functional sites, as shown in Figure 9a, we observe noticeable developments in vibronic features, which are responsible for the stretching modes of the naphthalene ring (bending ν_(NDC ring)_ at 778 cm^–1^) and metal oxide nodes (ν_(Li_^+^_–O)_ at 802). For instance, we observe an ∼8 cm^–1^ vibronic frequency shift in the aromatic ring bending mode of Li-NDC, centered at 778 cm^–1^ to the lower frequency while keeping the intensity constant. We associate this noticeable change to the binding of EC’s carbonyl oxygen with the Li^+^ metal node in the framework, causing a slight distortion in the vibronic bending modes of naphthalene rings. The reduction in intensity along with slight frequency shift (ν_(Li_^+^_–O)_ from 802 to 804 cm^–1^) observed for the Li^+^-O stretching mode of the metal oxide nodes reflects the mobile Li^+^ binding to the metal oxide nodes’ oxygens. These binding interactions and associated vibronic stretching frequencies are depicted in the structural representation in Figure 9a right.

The evidence for the binding of EC and LiClO_4_ on to the framework’s carbonyl groups can be identified from the frequency ranges of 1300–1450 and 1500–1650 cm^–1^. As shown in Figure 9b, in the frequency region of 1300–1450 cm^–1^, the mobile Li-ions exhibit cooperative binding interactions with the framework carboxylate oxygens, resulting in the disappearance of the ν_(C–O)_ stretch at 1341 and 1379 cm^–1^ while shifting the stretching frequency of the framework’s C–O–Li^+^ bonding mode at 1397–1391 cm^–1^ (also shown in the structural representation in Figure 9b). An additional cooperative interactions of the framework’s binding sites with the lithium salt as well as with EC’s carbonyl oxygen can be located from the NDC’s carbonyl stretching bands at 1542, 1567, and 1574 cm^–1^ and EC’s CH_2_ bending mode band at 1554 cm^–1^, respectively. The vibronic band at 1542 cm^–1^ in pure Li-NDC microstructures is less prominent in LEC@Li-NDC MOF and features a poorly resolved shoulder, which merges with the EC’s CH_2_ bending mode band centered at 1554 cm^–1^, confirming shared interactions of EC’s carbonyl oxygen with Li^+^ in LiClO_4_ as well as Li^+^ metal node in the Li-NDC framework (see the structural representation in Figure 9c right). The broadening and reduction in absorption intensity of the naphthalene ring’s ν_(C=O–Li_^+^_)_ band ranged from 1567 to 1574 cm^–1^ further convinces the additional binding to Li^+^ in the salt.^70,71^

Deviating from highly cooperative binding interactions present in LEC@Li-BDC, favoring both the framework’s functional sites and EC, the vibronic features ascribed from the FTIR spectral traces of the LEC@Li-NDC pellet confirm that LiClO_4_ binds to the active sites of the Li-NDC framework over EC’s carbonate group. Lack of strong binding of Li^+^ and ClO_4_^–^ onto EC also implies that guest molecules of LiClO_4_ and EC reside at a distance within the pores as Li-NDC possesses the largest pore volume. Thus, the results suggest that the Li-NDC MOF’s framework functional sites act as Li^+^ carrier sites, favoring the Li^+^ mobility along the framework edges over hopping through the porous channels. The largest pore volume, which hinders the binding of Li^+^ with the pore-filled EC due to the large intermolecular distance between the lithium salt and EC, rationalizes the Li^+^ conduction through the framework.

Comparing to the Li^+^ interactions in LEC@Li-BDC MOF and LEC@Li-NDC MOF, the vibronic features deduced from the FTIR spectral traces of LEC@Li-BPDC MOF pellets reveal that Li^+^ favors binding to EC’s active sites over the framework functional sites and EC acts as a bridge between the salt and MOF, by interacting with metal-ion nodes of the Li-BPDC framework (Figure 10). The noticeable frequency shift in pure EC’s ring bending stretch at 710–715 cm^–1^ along with the intensity reduction of the secondary band at 686 cm^–1^ provides a clear proof of Li^+^ binding to EC’s ether oxygen (Figure 10a). Moreover, the appearance of a weak shoulder peak (ν = 903 cm^–1^) on the high frequency side of the EC’s ring breathing band at 890 cm^–1^ (Figure 10b) and the significant frequency shifts of EC’s C–O–C stretching modes at 1058 and 1145 cm^–1^ to 1068 and 1158 cm^–1^, respectively, in the FTIR spectrum of LEC@Li-BPDC (Figure 10c) also supports the EC’s interaction with LiClO_4_. In terms of the salt interactions with the metal oxide nodes of the Li-BPDC framework, the frequency region from 725 to 850 cm^–1^ in the FTIR spectrum of LEC@Li-BPDC exhibits no changes to both the aromatic ring bending mode at ν = 772 cm^–1^ and the Li–O stretching mode for metal oxide nodes at ν = 840 cm^–1^, evidencing minimal interactions of mobile Li^+^ ions with the framework metal oxide nodes (Figure 10b). With the salt and EC, the stretching and combination band features for the vibronic modes of the organic linker carbonyls keep the same positions with no changes in absorption intensities. Instead, EC’s carbonyl group and ether oxygen bind to Li^+^ metal nodes in the framework, causing the reduction in the absorption intensities of the stretching bands for ECs CH_2_ scissoring at 1471 and 1484 cm^–1^ and EC’s CH_2_ bending at 1554 cm^–1^ (Figure 10d).^68,69^ The slight shift observed in the Li-BPDC MOF’s C–O stretch at 1397–1390 cm^–1^ provides additional evidence, supporting the EC’s interactions with the metal nodes. The structural representations on these binding interactions between an asymmetric unit of Li-BPDC and EC and the salt and EC are depicted in Figure 10e, along with associated vibronic modes and frequencies. Overall, our FTIR data on LEC@Li-BPDC MOF conclude that there is no binding of the lithium salt (either Li^+^ or ClO_4_^–^) onto the framework functional sites, confirming that, in LEC@ Li-BPDC, Li-ions move through the porous channels with the aid of only EC active sites. Thus, the EC acts as Li^+^ carrier sites, facilitating Li^+^ to move through the porous channels.

**Figure 11:**
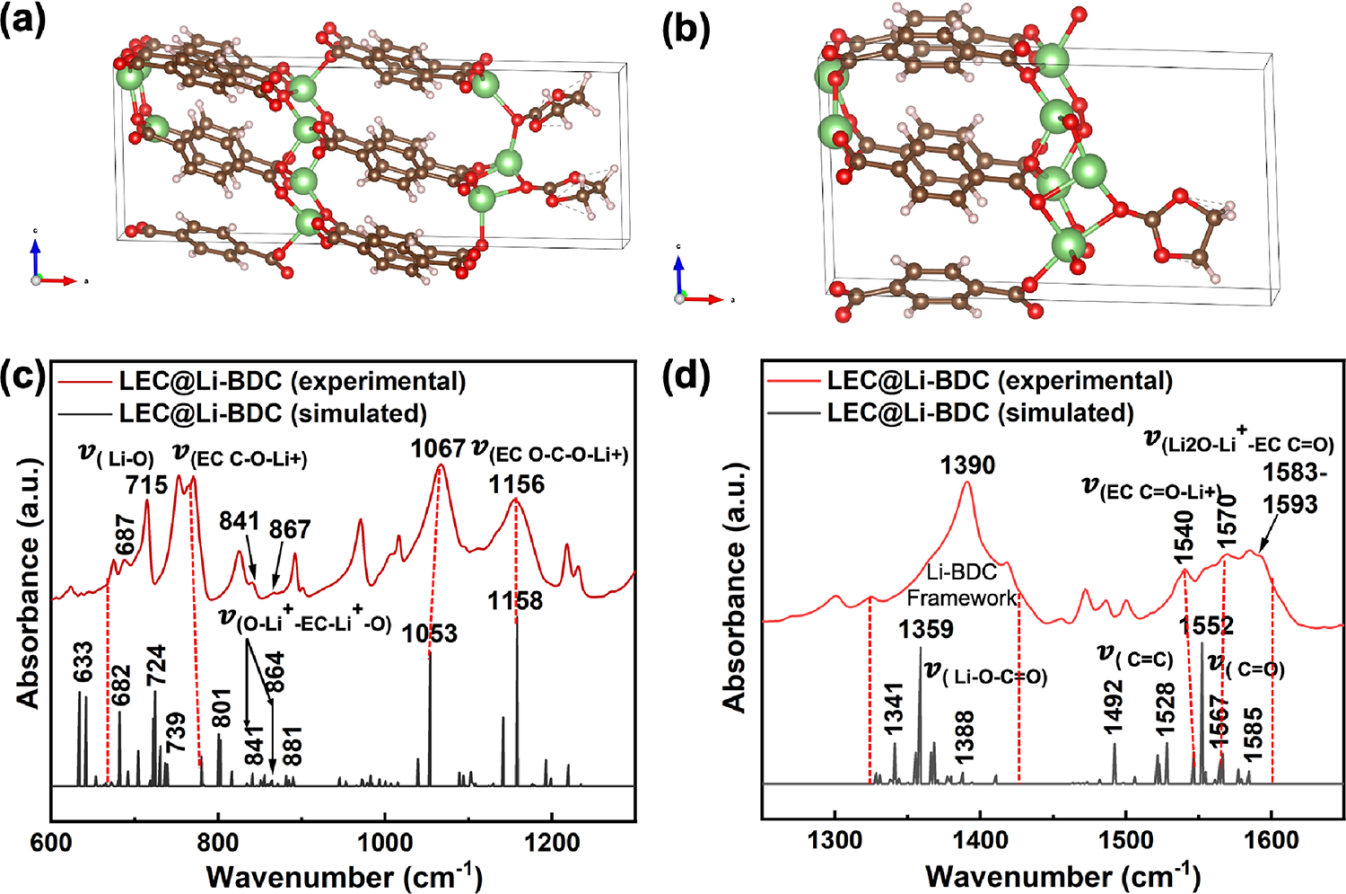
Optimized Li-BDC MOF structures with Li^+^ and EC, representing binding interactions to form two bridging complexes: (a) EC-Li^+^-EC complex and (b) Li_2_O-Li^+^-EC complex; and (c, d) experimental and simulated IR spectral traces of LEC@Li-BDC MOF of the regions, representing the binding interactions between the carbonyl group of EC and bound Li^+^ onto the framework carbonyl oxygen, and the regions, reflecting the binding interactions of lithium metal oxide noes (Li_2_O) with Li^+^ and EC’s carbonyl group.

The binding interactions deduced from experimental absorption spectra were validated by computational methods. The optimized Li-MOF structures were further subjected to structural optimization with lithium salt and EC filling into the framework’s cavities. As shown in Figure 11a,b, the optimized Li-BDC MOF structure with EC and Li^+^ ions agree the binding of EC and Li^+^ onto the framework’s Li_2_O metal oxide nodes and carbonyl oxygens, respectively. The simulated FTIR spectrum of LEC@Li-BDC (Figure 11c) confirms the formation of two bridging complexes, EC-Li^+^-EC and Li_2_O-Li^+^-EC, by interacting EC’s carbonyl group with Li^+^-O (ν = 841–864 and 1147–1152 cm^–1^), EC’s ether group with Li^+^ at the vibronic frequencies of ν = 724–739, 1053, and 1158 cm^–1^, and EC’s carbonyl group with Li_2_O-Li^+^ at ν = 1585 cm^–1^ (Figure 11d–e). These assigned vibronic frequencies support our comprehensive discussion on the experimental FTIR spectrum of the LEC@Li-BDC MOF.

**Figure 12:**
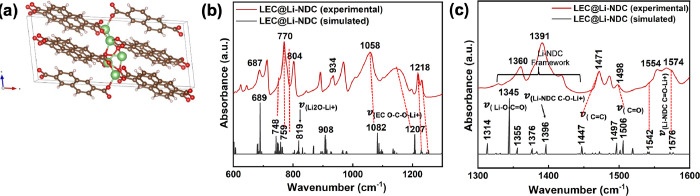
(a) Optimized Li-NDC MOF structure with Li^+^ representing the binding interaction between Li^+^ and Li_2_O metal oxide nodes and (b, c) the simulated and experimental IR spectral traces of LEC@Li-NDC (regions showing vibronic peaks corresponded to Li^+^ and LEC interactions with functional groups of Li-NDC).

As we observed from the experimental absorption spectrum of LEC@NDC, the most prominent interactions are the framework’s metal oxide nodes with mobile Li^+^ ions; we optimized the Li-NDC structure by introducing mobile Li^+^ onto the structure. Figure 12 a depicts the computationally optimized Li-NDC structure, with a free Li^+^ ions. The Li_2_O metal oxide nodes exhibit strong interactions with free Li^+^ ions, forming a bridging complex (Li_2_O-Li^+^-OLi_2_) with the framework. The simulated IR spectral traces compared with the respective regions of the experimental FTIR spectral traces are depicted in Figure 12b,c. The vibronic features of the optimized structure with the respective interactions between Li-NDC and Li-ions agree well with the experimental absorption spectral traces (Figure 12c). The prominent vibronic frequencies of the simulated spectrum at ν = 819 and 1542–1576 cm^–1^ (Figure 12b) are well correlated with the experimental vibronic frequencies at ν = 804 and 1567–1574 cm^–1^.

**Figure 13:**
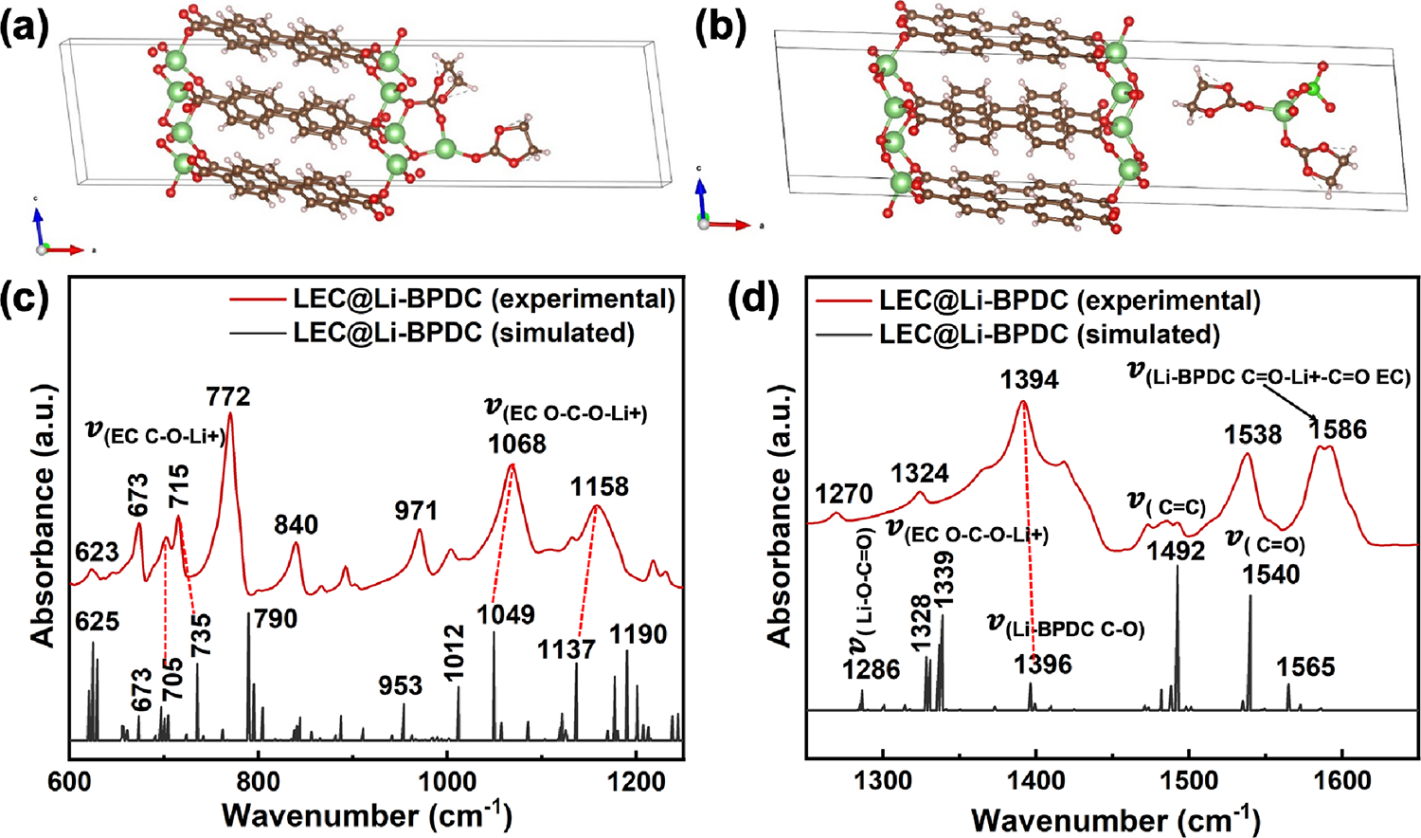
Optimized Li-BPDC MOF structure with (a) Li^+^ and EC and (b) Li^+^, EC, and ClO_4_^–^; and (c, d) the simulated IR spectrum of LEC@BPDC, representing binding interactions with EC, Li^+^, and ClO_4_^–^ that yield two bridging complexes Li_2_O-EC-Li^+^-EC and EC-Li^+^-EC-ClO_4_^–^ compared with the respective frequency region of the experimental FTIR spectral traces.

The Li-BPDC structures, optimized by introducing Li^+^, ClO_4_^–^, and EC (Figure 13a,b), confirm the binding interactions of the framework’s metal oxide nodes with Li^+^ ions and EC, forming two types of bridging complexes: Li_2_O-EC-Li^+^-EC and EC-Li^+^-EC-ClO_4_^–^. The simulated IR spectral results compared with the experimental IR data (Figure 13c,d) support our experimental findings on how mobile Li^+^ ions and EC interact with the framework of Li-BPDC to form these two bridging complexes. The corresponding vibronic frequencies of the simulated IR spectrum at ν = 705 and 735 cm^–1^ along with ν = 1049 and 1137 cm^–1^ confirm the interactions between ether oxygen of EC with free Li^+^, and ν = 1565 and 1584 cm^–1^ reflect the interactions of EC’s carbonyl group and the framework’s carbonyl group with Li^+^, respectively.

Based on the binding interactions deduced from the experimental and simulated absorption spectral traces of LEC@Li-MOFs, we can now associate our results to postulate the Li^+^ ion conduction mechanism for LEC@Li-MOF electrolytes and validate the mechanisms by elucidating the Li^+^ conduction pathway using the computation methods. To date, the solid electrolytes such as inorganic and polymer electrolytes exhibit Li^+^ ion hopping mechanisms, which either follow hopping from one coordination site to the vacant nearest site in inorganic solid electrolytes^11,56^ or follow the segmental motion of the polymer chains, inducing the ligand exchange of Li^+^ and Li^+^ hopping in polymer electrolytes.^11^ In MOF-based SSEs, prior research has shown that Li^+^ transport in the framework channels involves complex interactions between cations, anions, and framework segments.^56^ For example, a free motion of Li^+^ conduction mechanism involving cationic and anionic interactions has been revealed in MOFs by creating anionic channels via post modifications of OMCs of the MOF framework with anions like ClO_4_^–^, BF_4_^–^, NO_3_^–^, Cl^–^, and Br^–^.^14^ Additionally, in a recent study, adapting an open-pore conformation concept, free and bound states of Li^+^ conduction mechanism for the transfer of Li^+^ through nanochannels of metallacarborane (CoD^–^) ions incorporated into the MOF framework by post synthetic modification were demonstrated.^56^ Augmenting a similar approach of free and bound states of Li^+^ conduction through porous channels, which are preside by the isoreticular framework expansion of Li-MOFs, our findings strongly suggest that reticularly tailored pore channels and the framework functional sites cooperatively participate in a pore-filling-driven Li^+^ conduction mechanism. The coordination sites of the metal oxide nodes and metal-carboxylate segments in the framework edges and the plasticizer (EC) occupied in the pores enable Li^+^ ions to move through the porous channels. It is evident that, in LEC@Li-MOFs, depending on the pore volume, crystallite size, and the arrangement of the accessible pore channels, lithium salt interacts differently with the plasticizer and the framework’s functional sites, suggesting that the pore filling mechanism of Li^+^ ion transport in Li-MOFs differs from each other.

**Figure 14:**
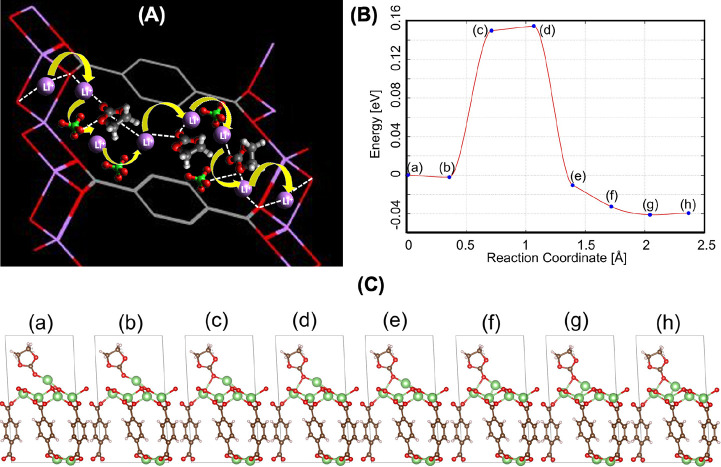
(A) Postulated Li^+^ conduction mechanism in LEC@Li-BDC based on the experimental and simulated absorption spectral traces, (B) the simulated Li^+^ migration energy profile for the bound state of the Li^+^ diffusion pathway in LEC@Li-BDC, and (C) the snapshots of corresponding conformational changes for the Li^+^ transport pathway in LEC@Li-BDC.

In the case of LEC@Li-BDC MOF, the experimental and simulated FTIR data provide direct evidence that the Li^+^ conduction mechanism follows the formation of Li^+^ bound states with the framework metal oxide nodes and pore-filled ECs, creating two different bridging complexes: Li_2_O-Li^+^-EC and EC-Li^+^-EC. As shown in Figure 14A, the Li^+^ bound state of Li_2_O-Li^+^-EC aids the Li^+^ from unit cell to unit cell in the crystal lattice, providing closely placed coordination sites for the Li^+^ from the framework edges to the pore channels. The second Li^+^ bound state bridging complex, i.e., EC-Li^+^-EC, which carries Li^+^ through the porous channels by hopping from one EC to another, building EC-Li^+^-EC bridges, facilitates the Li^+^ diffusion from pore to pore. In this mechanism, Li^+^ ions move as a single-ion bound state, with no movement of counteranions observed. Therefore, the LEC@Li-BDC electrolyte could be an ideal candidate for a single-ion solid-state conductor. However, the over binding between Li^+^ and the framework could lead to less favorable Li^+^ hopping with decreased mobility thereby lowering the ionic conductivity. This could be another reason that we observed the lowest ionic conductivity for LEC@Li-BDC. Additionally, it is now clear that, in LEC@Li-BDC, Li^+^ ion movement hinders by binding onto the framework while creating a higher grain boundary resistance in the material due to somewhat smaller crystallite size.^64^ Thus, the Li^+^ ions move as Li^+^ bound states, resulting in higher energy barrier for Li^+^ hoping through the porous network rather than moving in free ion-state through the porous channels.^57^

The above postulated Li^+^ conduction pathway by pore filling of lithium salt and EC into pores followed by formation of the Li^+^-bound state was elucidated using computational methods. The Li^+^ diffusion energy profile with respect to the confirmational changes of the Li^+^ bound state with EC and the Li_2_O metal oxide nodes during the movement of Li^+^ ions was obtained. As shown in Figure 14B, the Li^+^ ion moves by interacting with Li_2_O nodes of the Li-BDC framework and requires overcoming a Li^+^ migration barrier of 0.155 eV for a favorable Li^+^ movement through the framework by interacting with EC and the metal oxide nodes. The snapshots of conformational changes (Figure 14C), during the Li^+^ movement through the framework’s metal oxide nodes, reveal that the facile mobility of Li^+^ ions benefits from the binding interactions with the carbonyl oxygen of ethylene carbonate thereby stabilizing the movement of Li^+^ from one crystallite site to another crystallite site along the way of solid lattice, validating our postulated pore filling mechanism of Li^+^ movement by creating two bridgingcomplexes. (See the snapshot of Li^+^ movement within the framework of Li-BDC MOF in Figure S11).

**Figure 15:**
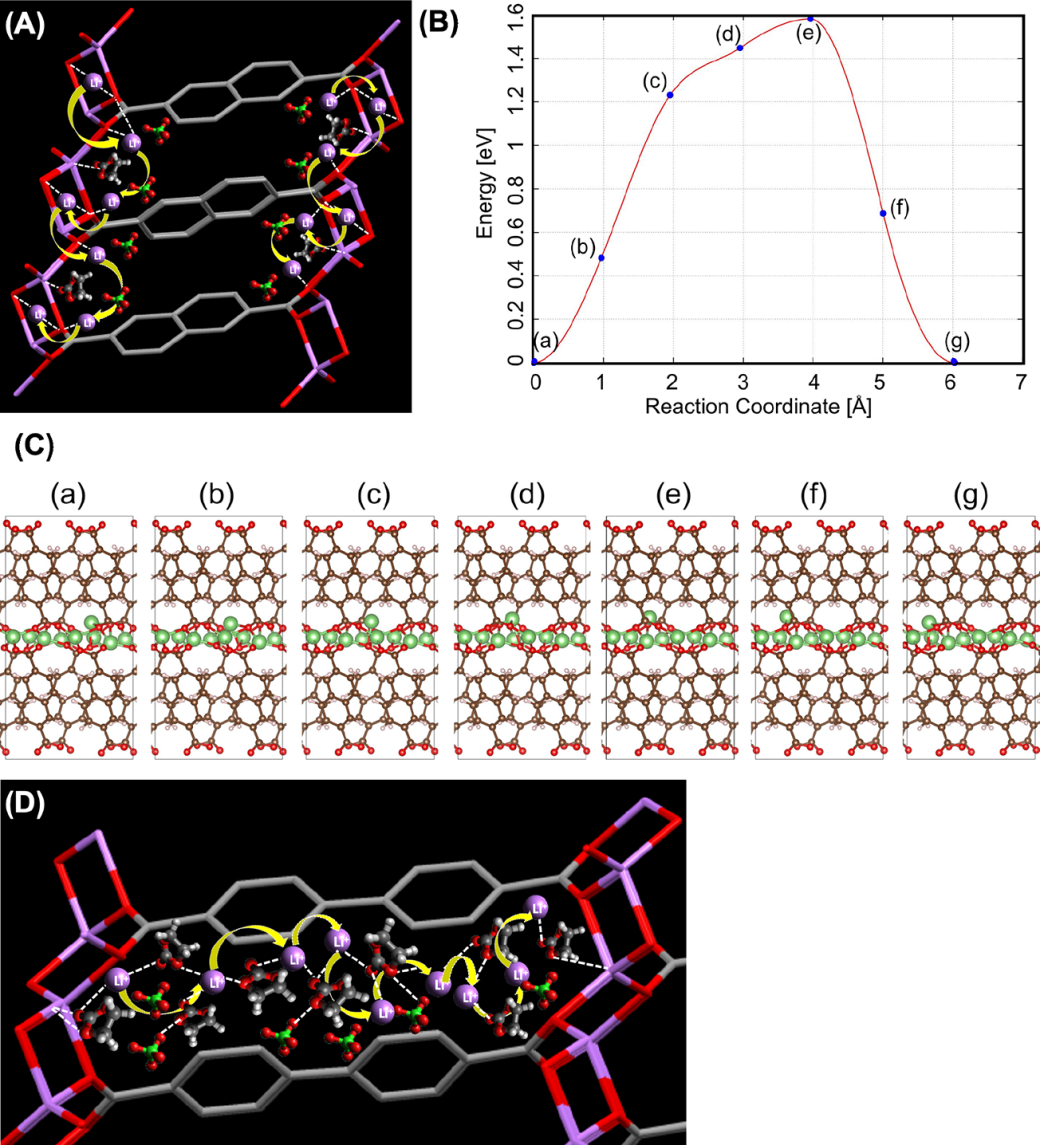
(A) Postulated Li^+^ conduction mechanism in LEC@Li-NDC based on the experimental and simulated absorption spectral traces, (B) the simulated Li^+^ migration energy profile for the bound state of the Li^+^ diffusion pathway in LEC@Li-NDC, (C) the snapshots of corresponding conformational changes for the Li^+^ transport pathway in LEC@Li-NDC, and (D) the postulated Li^+^ conduction mechanism in LEC@Li-BPDC based on the simulated and experimental IR spectra.

Abetting with the largest pore volume in Li-NDC, the experimental and simulated IR spectral analysis provide direct evidence for the presence of strong Li^+^ binding interactions with the framework coordination sites, suggesting the Li^+^ conduction along the framework edges and pore edges. Thus, we can rationalize that the Li^+^ conduction mechanism augments the Li^+^ hopping mechanism by forming Li^+^ bound states with the framework’s metal oxide nodes and carboxylate groups. As depicted in Figure 15A, Li^+^ ions bind to the metal oxide nodes’ oxygens and framework carboxylate coordination sites, yielding two different Li^+^ bound state complexes: [Li_2_O-Li^+^-OLi_2_] and [COO-Li^+^-OOC], which act as Li^+^ carriers and aid the Li^+^ hopping from one coordination site to another along the edges of the framework and the pore aperture. The weakly bound EC onto the framework carboxylate groups may aid hoping free Li^+^ interacting with the framework coordination sites, facilitating the Li^+^ migration through the framework edges. This could be a reason for observing a slightly higher ionic conductivity in the LEC@Li-NDC MOF compared to the LEC@Li-BDC MOF for the Li^+^ ion conduction.

As evidenced by the simulated and experimental absorption spectra, which confirm strong Li^+^ interactions with the framework coordination sites, we conducted computational studies for the optimized structure of Li-NDC in the presence of free Li^+^ and validated the Li^+^ conduction pathway. The energy profile for the bound state of Li-ions’ migration forming the [Li_2_O-Li^+^-OLi_2_] complex is shown in Figure 15B along with the respective snapshots of the conformational changes (Figure 15C) and the snapshot of Li^+^ movement (Figure S11). The computed energy profile confirms that the Li^+^ migration energy barrier, i.e., 1.58 eV, is considerably higher in the LEC@Li-NDC MOF compared to the computed energy barrier in LEC@Li-BDC. The difference in the energy barrier for Li^+^ migration further suggests that the movement of Li^+^ in each electrolyte depends on the pore volume and crystallite size, although both electrolytes follow the pore filling mechanism that involves Li^+^ bound states to move Li^+^ from one crystallite to another.

Agreeing with experimental IR spectral results, the computationally generated IR spectrum of LEC@Li-BPDC confirms that Li^+^ ions favor binding to the plasticizer while interacting with the framework’s carboxylate groups. The formation of two bridging complexes (Li_2_O-EC-Li^+^-EC and EC-Li^+^-EC-ClO_4_^–^) involving EC as a carrier for Li^+^ suggests “a vehicle-type” Li^+^ conduction mechanism, transporting Li^+^ through the porous channels. The experimental and theoretical vibronic stretching modes in LEC@Li-BPDC further suggest that our Li^+^ conduction mechanism may augment a cooperative mechanism, which involves both the ion hopping and solid-phase vehicle mechanism.^58,59^ The minimal binding of Li^+^ ions and the favorable binding of EC with the framework strongly evidence that the decoupling of Li^+^ transport through the framework segments, favoring both the ion hopping and vehicle mechanism.^58^ It is known that both the ion hopping and vehicle mechanisms can allow for the decoupling of ion motion from the host fragments.^72,73^ Thus, based on our findings from the FTIR spectral elucidation, the Li^+^ conduction mechanism in LEC@Li-BPDC creates a long-range order continuous Li^+^ hopping pathway by bridging EC molecules with the framework Li^+^-ion metal node and the pore-filled Li^+^ ions and ClO_4_^–^, facilitating Li^+^ hopping from one EC molecule to another through the porous channels.

As depicted in Figure 15D, pore-filled EC binds to the Li^+^ of the metal oxide nodes (Li_2_O) in the framework edges and to the lithium salts occupied in the pores, creating a continuous chain of (Li_2_O-EC-Li^+^-EC−) and (-EC-Li^+^-EC-ClO_4_^–^) bridging complexes. The bound states of Li^+^ in these bridging complexes hop from one coordination site to another while influencing to generate free volume for the mobility of free states Li^+^ ions through the pore channels, resulting in higher ionic conductivity. However, we were unable to obtain the energy profile along with respective conformational structures for simulating the postulated Li^+^ migration pathway from the computational studies due to considerably two larger bridging complexes involving multiple ionic species. Regardless, the optimized structures for two bridging complexes along with the simulated IR spectral traces strongly support our postulated mechanism, which transfers Li^+^ via cooperative ion hopping and vehicle-type conduction.

## 4 Conclusions

In summary, we have established an analytical foundation to design high-performance solid and quasi-solid electrolytes from isoreticular MOFs, by providing a microscopic picture of the Li^+^ conduction mechanism in isostructural Li-MOFs. The outcome of our study shows that the cooperative function of reticularly tailored pore volume, with an average micropore width of 18.59 Å, the long-rage order of extended framework structure with length of 13.52 Å, and crystallite size (11.78 nm) of Li-BPDC MOF, facilitates the Li-ion conduction, thereby enhancing the Li^+^ conductivity in the solid-state at room temperature. The textural results of Li-MOFs confirm that the pore volume does not necessarily follow the extended framework structure with respect to linker length (vertices lengths) but rather results in an optimal pore volume with the long-range order of the framework structure and the bimodal porosity distribution. The gradual decrease in crystallite size and pore volume promotes the Li^+^ conductivity in the order of Li-BDC < Li-NDC < Li-BPDC in LEC@Li-MOFs. We find that ionic conductivities of LEC@Li-MOFs at room temperature are in the comparable range with the ionic conductivities of current state-of-the-art solid polymer electrolytes. However, the downside is the somewhat high activation energies observed for our electrolytes because of their Li^+^ conduction mechanism, which involves both free and bound states of Li^+^ in the ion transport process. With strong support from computational methods, we found that our electrolytes follow a pore filling-driven Li^+^ conduction mechanism, which involves movement of both free and bound states of Li^+^ via an ion hopping-type mechanism or both ion hopping and vehicle-type mechanisms. The ion hopping mechanism involves Li^+^ bound states, forming different bridging complexes with the framework’s functional sites and the pore-filled plasticizer’s active sites, and aids the Li^+^ hopping from one coordination site to another. The proposed solid-phase vehicle mechanism is supported by the pore-filled ethylene carbonate, which acts as a vehicle, carrying Li^+^ through the porous channels. Our results highlight the importance of the reticular design of MOFs as a powerful tool to understand the solid-phase Li-ion conductivity in MOFs. The insight presented here with the following key design guidelines could enable further accelerating the development of the next generation of high-performance solid ionic conductors.

The key design guidelines revealed from our results are:

1. Select MOFs with extended framework structure, having a narrower pore volume (≤0.618 cm^3^/g), smaller pore width (≤18.6 Å), and rather small crystallite size (≤12.0 nm) to promote Li-ion conduction via pore filling mechanisms, involving bridging complexes for ion hopping and vehicle-type ion transfer from one crystallite site to another.
2. Design solid electrolytes by encapsulating lithium salt and the plasticizer with MOFs, having fully coordinated metal-ion nodes (none-open metal centers) to promote binding interactions of framework functional sites with Li^+^ and the plasticizer.
3. Prescreen MOFs using computational methods to deduce the Li^+^ diffusion energy barrier for the pore filling mechanism, introduced in this work, using the Li^+^ bound state bridging complexes.

## Supporting Information Available

Bulk elemental analysis, FTIR spectra, TGA, crystal lattice planes, and BET isotherms of Li-MOFs are included and snapshots of Li^+^ movement in Li-BDC and LI-NDC MOFs (PDF)
A video clip on the computationally simulated Li-ion diffusion pathway in Li-BDC MOF (MP4)
A video clip on the computationally simulated Li-ion diffusion pathway in Li-NDC MOF (MP4)
