# Supplementary material for: Insight into the Isoreticularity of Li-MOFs for the Design of Low-Density Solid and Quasi-Solid Electrolytes
Source: Chem Mater. 2023 Nov 28;35(23):9857–78. doi: 10.1021/acs.chemmater.3c01021 (PMC10720344; doi:10.1021/acs.chemmater.3c01021)
Supplement: Supplementary file 1 — cm3c01021_si_001.pdf [file cm3c01021_si_001.pdf]

Supporting Information

Insight into the Isoreticularity of Li-MOFs for the Design of Low-density Solid and Quasi-Solid Electrolytes

Pravalika Butreddy, Manoj Wijesingha, Selina Laws, Gayani Pathiraja, Yirong Mo, *and Hemali Rathnayake**

[1]*Department of Nanoscience, Joint School of Nanoscience and Nanoengineering, University of North Carolina at Greensboro, Greensboro, NC 27401.*

Table S1. Elemental compositions of Li-MOFs obtained from the bulk analysis of XPS elemental survey.

Li-MOFs	XPS Elemental Composition Experimental (wt%)			Elemental Composition Theoretical (wt%)			
	Li	C	O	Li	C	O	H
Li-BDC	7.87	55.98	36.15	7.80	53.98	35.95	2.27
Li-NDC	6.05	64.03	29.92	6.09	63.20	28.06	2.65
Li-BPDC	4.89	68.48	26.63	5.46	66.18	25.19	3.17

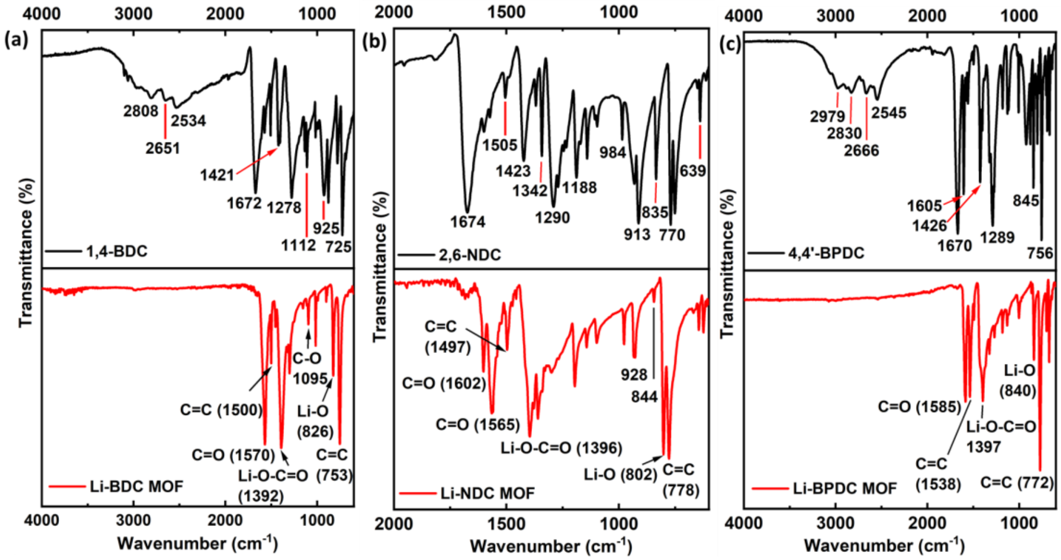

Figure S1. FTIR spectral traces of: (a) 1,4-BDC linker and Li-BDC MOF, (b)2,6-NDC linker and Li-NDC MOF (ULMOF-1), and (c) 4,4′-BPDC and Li-BPDC (ULMOF-2).

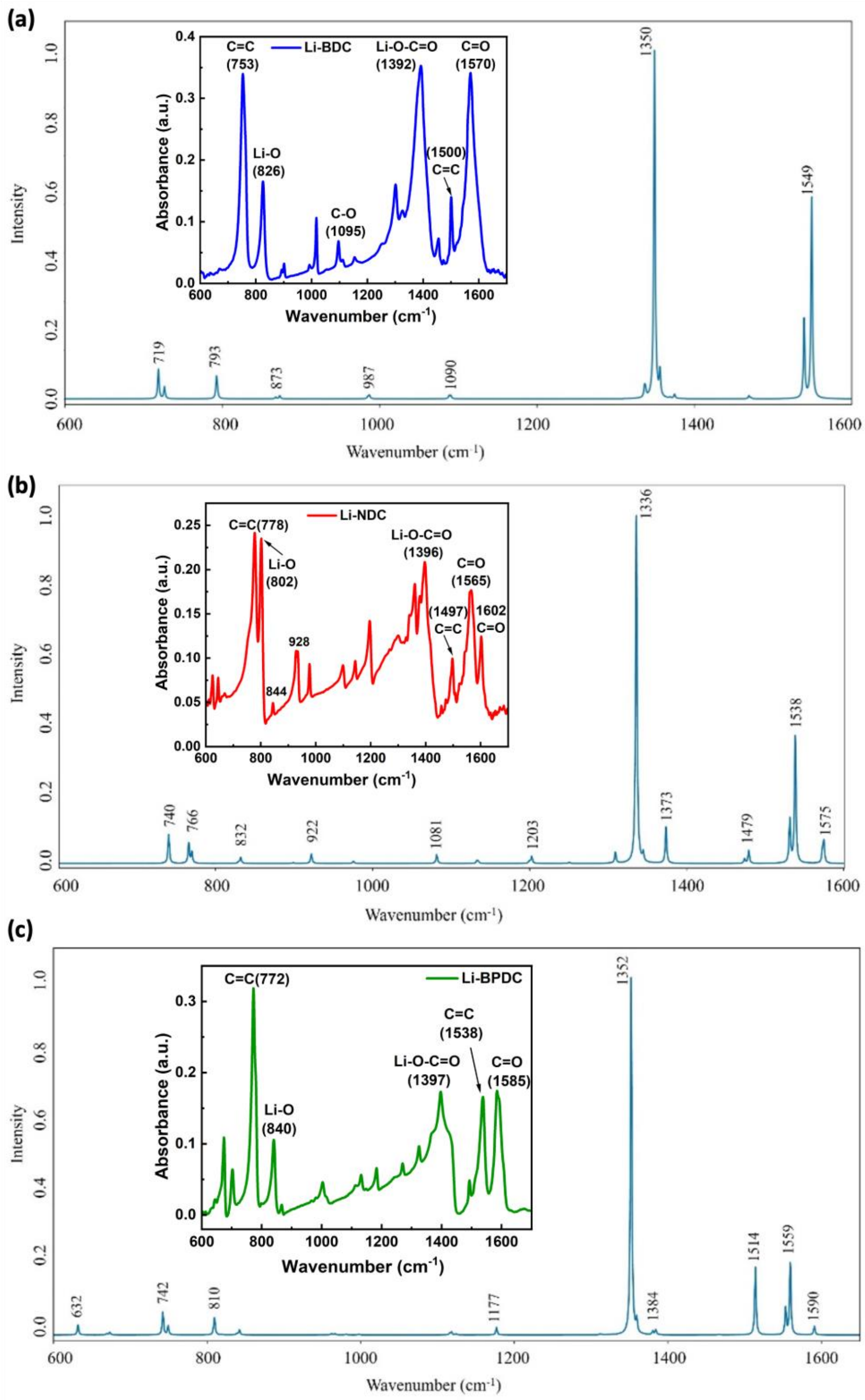

Figure S2: Simulated FTIR spectra of: (a) Li-BDC; (b) Li-NDC; and (c) Li-BPDC; Inset: Respective experimental FTIR spectra for the simulated region from 600 to 1700 cm^{-1}.

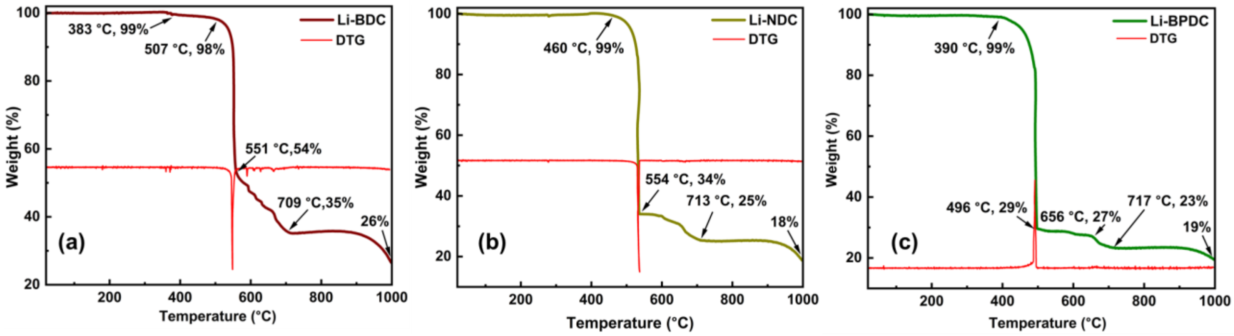

Figure S3. TGA Plots along with %weight losses of: (a) Li-BDC, (b) Li-NDC, and Li-BPDC.

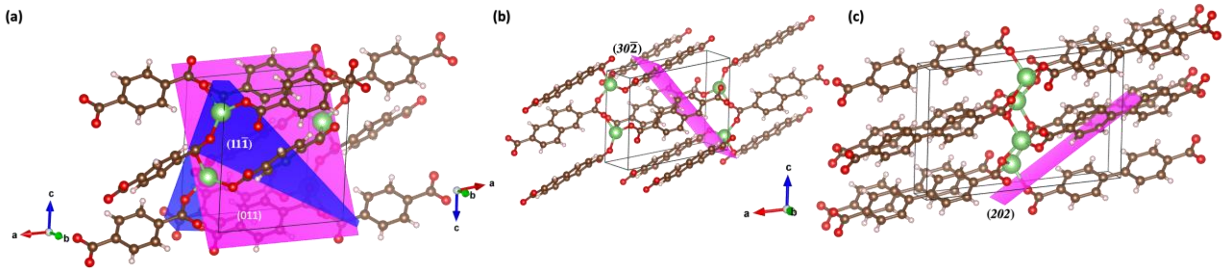

Figure S4. Crystal lattice planes corresponding to the lattice planes identified by HR-TEM for: (a) Li-BDC, (b) Li-NDC, (c) Li-BPDC.

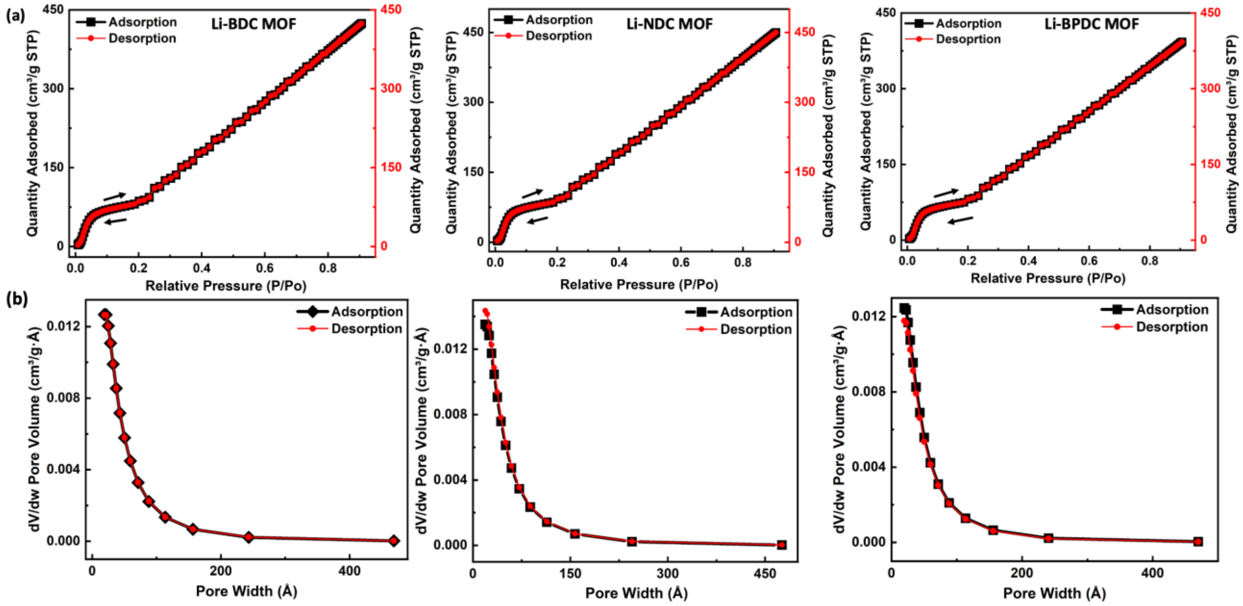

Figure S5. (a) BET Isotherms, and (b) BJH adsorption and desorption pore volume distribution plots for Li-MOFs.

Table S2: Elemental compositions of LEC@Li-MOFs obtained from the bulk analysis of XPS elemental survey.

LEC@-Li MOFs	XPS Elemental Composition Experimental (wt%)			XPS Elemental Composition Experimental (At%)		
	Li	C	O	Li	C	O
LEC@ Li-BDC	8.37	48.28	42.67	15.47	50.70	33.64
LEC@Li-NDC	7.55	51.38	40.51	11.60	55.11	32.61
LEC@Li-BPDC	5.39	42.56	48.68	8.74	46.83	40.22

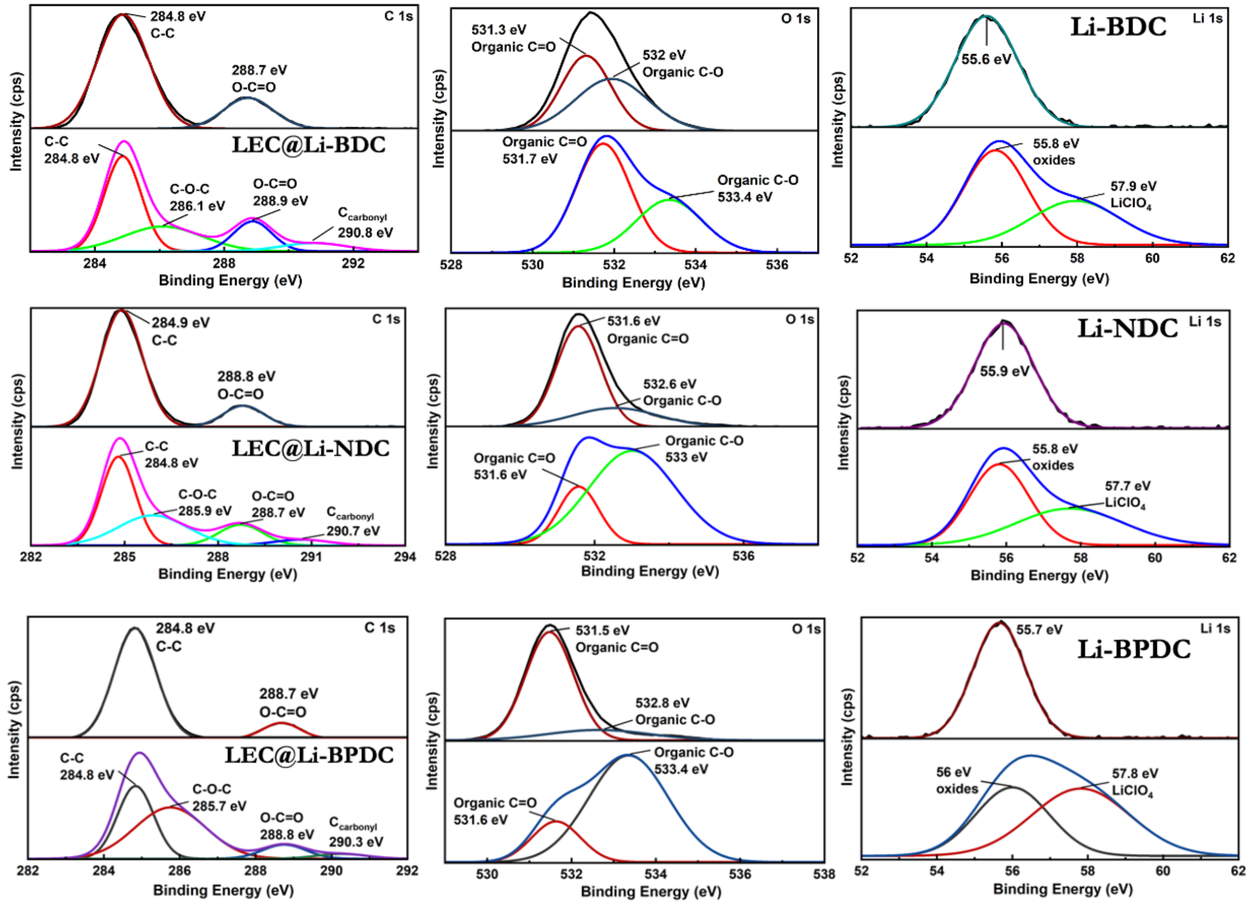

Figure S6: Binding energy spectra of (b) Li 1s, (c) C 1s, and (d) O 1s for LEC@Li-MOFs and pristine Li-MOFs.

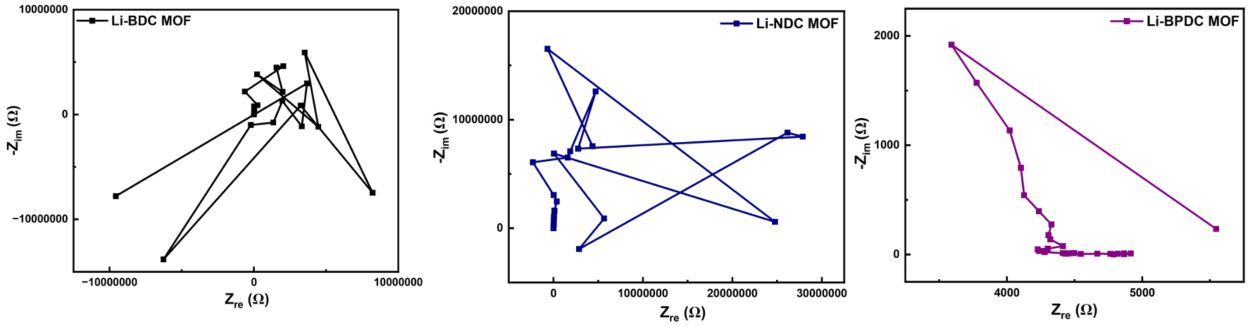

Figure S7: Nyquist plots at for pristine pellets of Li-BDC, Li-NDC, and Li-BPDC, representing open circuit current.

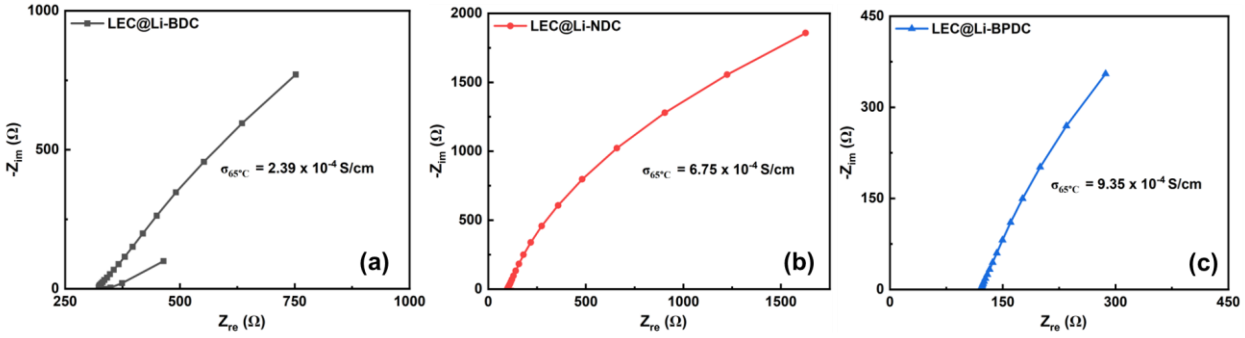

Figure S8. Nyquist plots at 65℃ for: (a) LEC@Li-BDC, (b) LEC@Li-NDC, and (c) LEC@Li-BPDC.

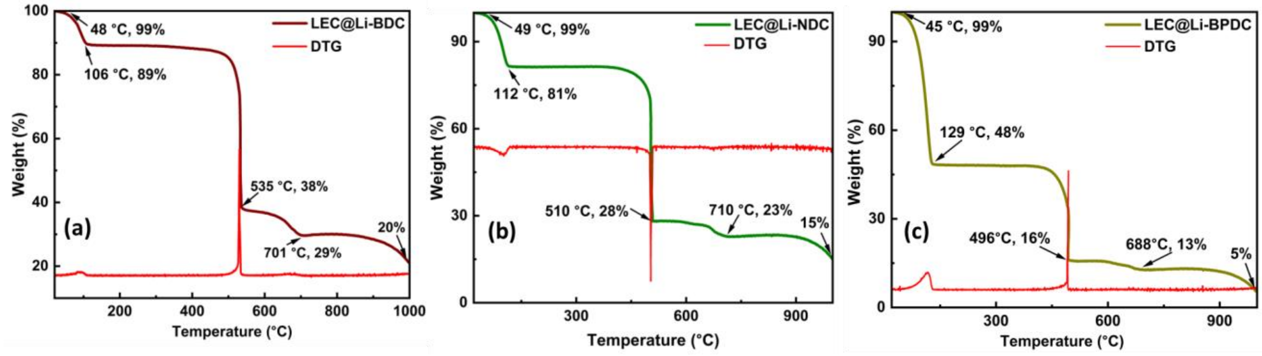

Figure S9: TGA graphs of LEC@Li-MOFs: (a) LEC@Li-BDC, (b) LEC@Li-NDC, and (c) LEC@Li-BPDC.

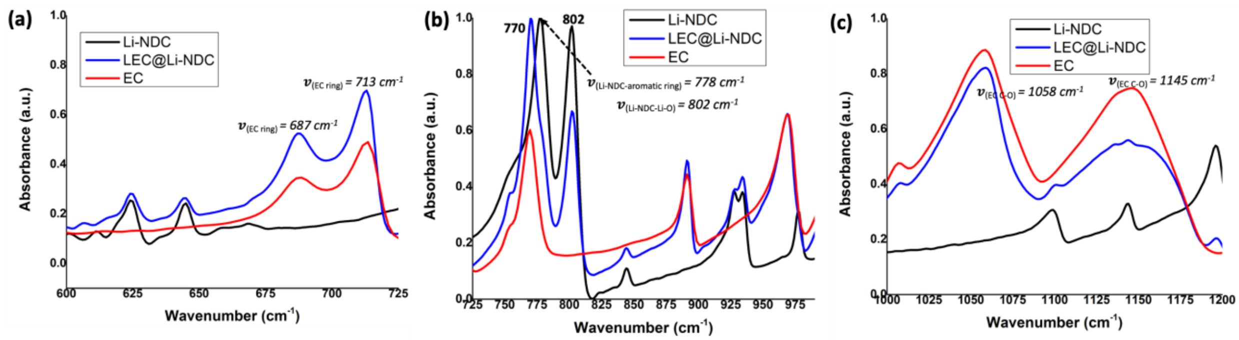

Figure S10. Comparison FTIR spectral traces of Li-NDC, LEC@Li-NDC, and EC for the spectral region of: (a) 600 cm^{-1} to 725 cm^{-1}; (b) 725 cm^{-1} to 975 cm^{-1}, and (c) 1000 cm^{-1} to 1200 cm^{-1}.

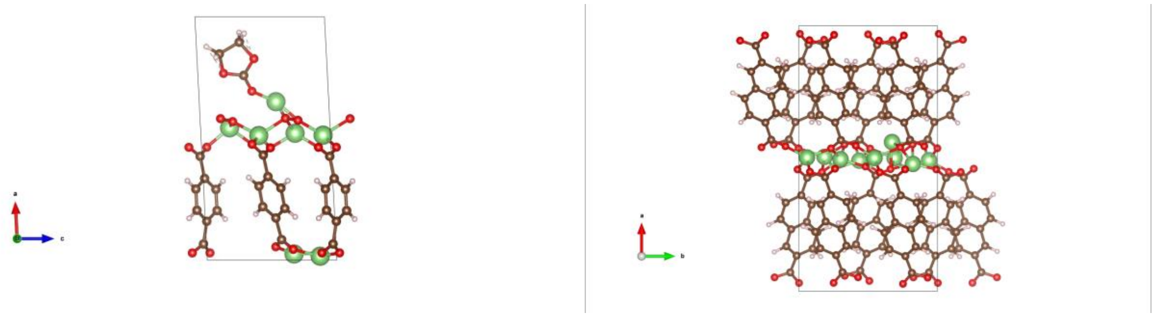

Figure S11. Movie clips of Li^+ movement in: Left- LEC@ Li-BDC MOF; and Right- LEC@Li-NDC MOF.
